# Improving the OER Activity of Titania Via Doping and Adlayers

**DOI:** 10.1002/open.202400085

**Published:** 2025-02-25

**Authors:** Anna Gomer, Thomas Bredow

**Affiliations:** ^1^ Mulliken Center for Theoretical Chemistry Clausius-Institut für Physikalische und Theoretische Chemie Universität Bonn Beringstraße 4 53115 Bonn Germany

**Keywords:** Oxygen Evolution Reaction, Overpotential, Adsorption, Density functional theory, Electrocatalytic water splitting

## Abstract

The oxygen evolution reaction (OER) was investigated theoretically on modified rutile(110) surfaces at density functional theory level in search for inexpensive but active catalyst materials required for water electrolysis. Ti substitution by Nb in rutile and furthermore adding adlayers of transition metal (TM) oxides, with TM= Ir, Ru and Rh, substantially improves titania OER activity. The catalytic activity was assessed by the overpotential of the OER which was calculated from adsorption energies of the intermediates M−O, M−OH and M−OOH. Different reaction mechanisms were suggested depending on the presence or absence of M−OOH. Materials with iridium dioxide in the top layer have similar overpotentials, both as adlayer on (doped) ${\mathrm{TiO}}_{2}$ and as pure ${\mathrm{IrO}}_{2}$ . Thus, the percentage of this expensive and scarce element can be drastically reduced without deteriorating the activity. A monolayer of ${\mathrm{RuO}}_{2}$ on rutile ${\mathrm{TiO}}_{2}$ has an even lower overpotential compared to pure ${\mathrm{RuO}}_{2}$ . In addition, ${\mathrm{RhO}}_{2}$ and ${\mathrm{RhO}}_{2}$ :${\mathrm{Nb}}_{1/3}{\mathrm{Ti}}_{2/3}O_{2}$ were identified as catalysts with higher OER activity than ${\mathrm{IrO}}_{2}$ .

## Graphical abstract

To improve the activity of the oxygen evolution reaction, materials based on TiO_2_‐rutile were doped or/and adlayers were added on the supporting material. Different reaction mechanisms were analyzed. A better overpotential than state‐of‐the‐art IrO_2_ was found.

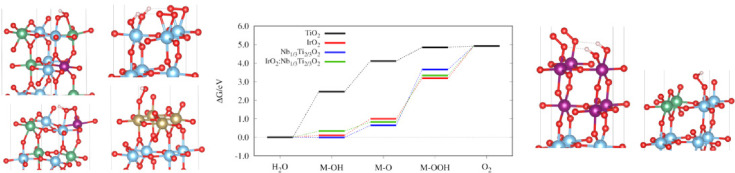

## Introduction

Population growth and increasing energy demand are driving global climate change due to carbon dioxide emissions. A critical technological challenge of the 21st century is thus the decarbonization of the global economy. An important contribution to this transition is the production of green hydrogen as a carbon‐neutral alternative to fossil fuels.^[1]^ Hydrogen production by chemical water splitting includes the oxygen evolution reaction (OER) and hydrogen evolution reaction (HER).[^2^, ^3^] The significantly slower kinetics of the OER determine the overall reaction rate and is the focus of our study. The state‐of‐the‐art electrocatalyst for the OER is ${\mathrm{IrO}}_{2}$ .^[4]^ However, the scarcity^[5]^ and high price of Ir call for alternatives.^[6]^ One possibility is to employ materials based on the more affordable titanium dioxide, which already has a plethora of applications,[^7^, ^8^, ^9^, ^10^, ^11^, ^12^, ^13^, ^14^, ^15^, ^16^] in electrocatalytic reactions^[17]^ and photocatalysis.[^18^, ^19^, ^20^] Rutile as the most stable polymorph of single‐crystal and microcrystalline ${\mathrm{TiO}}_{2}$ ^[21]^ has been chosen in our previous work^[22]^ and also in this study.

There are two options for improving catalyst activity, either increasing the number of catalytically active sites or increasing the intrinsic activity.^[23]^ In this work, we will focus on the latter, especially, since the improvement of the intrinsic activity reduces the catalyst costs. This approach has also been applied to the HER, where the improvement through increasing the intrinsic activity was orders of magnitude larger than the activity difference between high and low loading catalysts.^[23]^ There are numerous possibilities to tune the electrocatalytic activity and find an alternative for ${\mathrm{IrO}}_{2}$ . ${\mathrm{TiO}}_{2}$ is semiconducting[^24^, ^25^] and can e. g. be doped with other transition metals to increase its conductivity.^[26]^ A correlation between conductivity and OER activity has been previously observed.^[27]^ The approach of investigating various transition metal substitutions has also been successful, e.g. for organometallic structures.[^28^, ^29^] Additionally, adlayers of other transition metal dioxides can be added on pure or doped rutile to increase the activity or entirely different materials can be tested.

## Reaction Mechanisms

Different pathways have been proposed for the oxygen evolution reaction. In the following, the mechanisms with M−O, M−OH and M−OOH as intermediates are introduced, M representing the active metal site at the surface. The conventional mechanism of the OER is the adsorbate evolution mechanism (AEM)^[30]^ or mononuclear mechanism,^[31]^ which involves four concerted proton/electron transfer reactions on the metal ion according to Eqs. 1–4.[^32^, ^33^]

(1) $$H_{2}O(l)+M\to M-\mathrm{OH}+H^{+}+e^{-}$$

(2) $$M-\mathrm{OH}\to M-O+H^{+}+e^{-}$$

(3) $$M-O+H_{2}O(l)\to M-\mathrm{OOH}+H^{+}+e^{-}$$

(4) $$M-\mathrm{OOH}\to M+O_{2}\left(g\right)+H^{+}+e^{-}$$

A water molecule reacts with a coordinatively unsaturated metal atom M and forms a hydroxide M−OH on this coordinatively unsaturated site (CUS). After deprotonation, the oxygen atom on the surface reacts with another water molecule leading to M−OOH. The last step consists of the oxygen molecule formation and the generation of a free active site for the next reaction cycle.[^32^, ^33^] The reaction intermediates are M−O, M−OH and M−OOH, which are treated as the adsorbed species in our study.

The dissociative mechanism^[34]^ or electrochemical oxide path,^[35]^ represented by Eq. 5–7, has the same two first reaction steps as the AEM. However, the third step is the direct coupling of two M−O forming an oxygen molecule. In this reaction mechanism, the reaction intermediate M−OOH is not present.

(5) $$H_{2}O(l)+M\to M-\mathrm{OH}+H^{+}+e^{-}$$

(6) $$M-\mathrm{OH}\to M-O+H^{+}+e^{-}$$

(7) $$2M-O\to 2M+O_{2}\left(g\right)$$

In the bifunctional mechanism I^[31]^ described by Halck et al.,^[36]^ in addition to the surface metal atom M as the active site, a surface oxygen atom ${O^{*}}_{A}$ serves as a proton acceptor. The reaction intermediates are the deprotonated intermediates of AEM, compare Eqs. 8–11.

(8) $$H_{2}O(l)+M+O_{A}^{*}\to M-O+H-O_{A}^{*}+H^{+}+e^{-}$$

(9) $$M-O+H-O_{A}^{*}\to M-O+O_{A}^{*}+H^{+}+e^{-}$$

(10) $$H_{2}O(l)+M-O+O_{A}^{*}\to M-\mathrm{OO}+H-O_{A}^{*}+H^{+}+e^{-}$$

(11) $$M-\mathrm{OO}+H-O_{A}^{*}\to M+O_{A}^{*}+O_{2}\left(g\right)+H^{+}+e^{-}$$

There is also a variation of the first two steps of this mechanism discussed by Exner,^[31]^ in which the proton acceptor acts only in the second reaction step, see Eq. 12–13.

(12) $$H_{2}O(l)+M+O_{A}^{*}\to M-\mathrm{OH}+O_{A}^{*}+H^{+}+e^{-}$$

(13) $$M-\mathrm{OH}+O_{A}^{*}\to M-O+O_{A}^{*}+H^{+}+e^{-}$$

In the bifunctional mechanism II introduced by Exner,^[31]^ the oxygen surface atom acts also as a proton acceptor as in the bifunctional mechanism I, but the reaction intermediates are M−O, M−OH and M−OOH as in the AEM. This is due to the third reaction step in which water splitting yields M−OOH and $H-{O^{*}}_{A}$ . The deprotonation of ${O^{*}}_{A}$ is considered a separate reaction step, and the last step is the formation of $O_{2}$ from M−OOH as in the AEM, compare Eqs. 14–18.

(14) $$H_{2}O(l)+M+O_{A}^{*}\to M-\mathrm{OH}+O_{A}^{*}+H^{+}+e^{-}$$

(15) $$M-\mathrm{OH}+O_{A}^{*}\to M-O+O_{A}^{*}+H^{+}+e^{-}$$

(16) $$H_{2}O(l)+M-O+O_{A}^{*}\to M-\mathrm{OOH}+H-O_{A}^{*}$$

(17) $$H-O_{A}^{*}\to O_{A}^{*}+H^{+}+e^{-}$$

(18) $$M-\mathrm{OOH}\to M+O_{2}\left(g\right)+H^{+}+e^{-}$$

There is also a mechanism, in which the oxygen atoms of the surface are involved in the reaction.^[30]^ This lattice‐oxygen‐mediated mechanism (LOM) has no M−OOH as an intermediate. Two deprotonated M−OH on the metal sites result in two neighboring oxo species which directly form the O−O bond. Forming $O_{2}$ leaves two vacant metal centers, which are again occupied by $H_{2}O$ . However, the LOM cycle is considered to have a large activation barrier. Structure and crystallinity seem to be decisive for whether lattice oxygen exchange takes place.^[30]^ Nevertheless, the LOM cycle is not considered in this work because of the large activation barrier.^[30]^

There are other mechanisms in which two neighboring metal atoms act as adsorption sites. However, they are not the preferred pathways at most potentials^[31]^ and are therefore not considered in this study.

In many recent studies, the stability of surface reaction intermediates is calculated by applying density functional theory (DFT) methods, since it is not easily obtained experimentally.^[34]^ The adsorption energies ΔE of the intermediates are calculated with respect to the bare surface $E_{\mathrm{slab}}$ , water $E_{H}$ 2_O_ and hydrogen $E_{H}$ 2 in the gas phase,^[32]^ compare Eq. 19. In this way, the calculation of the electronic triplet ground state of molecular oxygen is avoided[^5^, ^37^, ^38^] since it is not accurately described with Generalized Gradient Approximation (GGA) functionals.[^39^, ^40^] Negative ΔE values indicate stabilization of the intermediate with respect to dissolution.

(19) $$\begin{array}{cc} {\Delta E}_{M-O}\; & =E_{M-O}-E_{\mathrm{slab}}-\left(E_{H_{2}O}-E_{H_{2}}\right)\;\\ {\Delta E}_{M-\mathrm{OH}}\; & =E_{M-\mathrm{OH}}-E_{\mathrm{slab}}-\left(E_{H_{2}O}-\frac{1}{2}E_{H_{2}}\right)\;\\ {\Delta E}_{M-\mathrm{OOH}}\; & =E_{M-\mathrm{OOH}}-E_{\mathrm{slab}}-\left(2E_{H_{2}O}-\frac{3}{2}E_{H_{2}}\right)\; \end{array}$$

To obtain a descriptor for the activity of the OER, the overpotential is calculated. It is derived from the Gibbs free energies of the individual reaction steps. In this work, similar to previous theoretical studies,[^32^, ^33^, ^36^, ^41^, ^42^, ^43^] no activation barriers but thermodynamic data were taken into account for the overpotential. Similarities between thermodynamic and kinetic volcano plots obtained from microkinetic modeling^[40]^ validate the use of a thermodynamic approach.^[44]^

The Gibbs free energy of the conventional AEM is calculated using Eq. 20., ^[32]^

(20) $$\begin{array}{cc} {\Delta G}_{1}^{\mathrm{OER}}\; & ={\Delta G}_{M-\mathrm{OH}}-eU+k_{B}T\ln a_{H^{+}}\;\\ {\Delta G}_{2}^{\mathrm{OER}}\; & ={\Delta G}_{M-O}-{\Delta G}_{M-\mathrm{OH}}-eU+k_{B}T\ln a_{H^{+}}\;\\ {\Delta G}_{3}^{\mathrm{OER}}\; & ={\Delta G}_{M-\mathrm{OOH}}-{\Delta G}_{O}-eU+k_{B}T\ln a_{H^{+}}\;\\ {\Delta G}_{4}^{\mathrm{OER}}\; & =4.92\mathrm{eV}-{\Delta G}_{M-\mathrm{OOH}}-eU+k_{B}T\ln a_{H^{+}}\; \end{array}$$

For the Gibbs free energy differences ${\Delta G}^{\mathrm{OER}}$ , the reference potential is considered to be that of the standard hydrogen electrode. The chemical potential $\mu_{H^{+}}$ of $H^{+}+e^{-}$ is related to that of 1/2 $H_{2}$ in such a way, that the free energies are the same at standard conditions (pH =0 , T= 298 K and U=0 , U being the external electrode potential). As a result, the last part of the equations vanishes leaving only the Gibbs free energies of the intermediates. Subsequently, the formation energy of $O_{2}$ (4.92 eV) is equal to the sum of the Gibbs free energies of the reaction steps.[^32^, ^33^, ^34^]

The theoretical overpotential for the OER $\eta^{\mathrm{OER}}$ introduced by García‐Mota et al.^[32]^ is defined in Eq. 21 as the highest ${\Delta G}_{\max}^{\mathrm{OER}}$ of the four reaction steps divided by the electron charge e and by subtracting 1/4 of the $O_{2}$ formation energy, 1.23 V.^[32]^

(21) $$\eta^{\mathrm{OER}}=\left({\Delta G}_{\max}^{\mathrm{OER}}/e\right)-1.23V$$

This theoretical overpotential of the AEM was used as an activity descriptor for the oxygen evolution reaction in our study. According to the Sabatier principle, an ideal catalyst should neither bind the reactant too strongly nor too weakly, so that the intermediates can bind and react, but the product can also desorb again.[^45^, ^46^] The ideal OER catalyst requires all free energies of the four reaction steps to have the same value, namely 1.23 V.^[30]^ Thus, the ideal overpotential should be zero.

## Results and Discussion

In our previous work, Sc, Nb, and Ta were identified as promising candidates for Ti substitution in rutile to obtain stable mixed oxides with sufficiently high conductivity. Among those, Ta and Nb are the most favorable due to metallic behavior for high dopant concentrations.^[47]^ For niobium, theory and experiment identified the highest conductivity for 33 % or 35 % substitution, respectively.^[48]^ The surface energies and the resulting equilibrium shapes predicted by the Wulff‐Gibbs theorem^[49]^ calculated with PBE−D4[^50^, ^51^, ^52^] were previously published.^[22]^ The thermodynamically most stable surface is (110) for pure and Nb‐ or Ta‐doped rutile. This surface was also considered in this work. The geometry optimization of the bulk yielded the lattice parameters that were employed for the construction of slab models. Our basic model for the (110) surface has six stoichiometric layers. For the adlayers, 8 layers were used and the Ti atoms of the topmost layers on both ends were substituted with the respective transition metal M.

The following result section is divided into Surface Optimizations, Adsorption, Frequency Calculations, Reaction Diagrams and Overpotential, Bader Charge Analysis and Band Structures.

## Surface Optimizations

**Figure 1:**
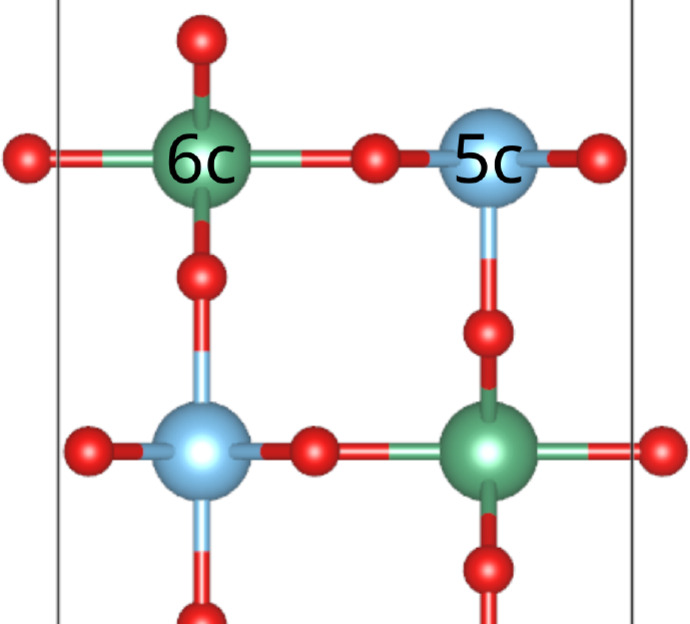
5c‐ and 6c‐sites of the metal atoms in $M_{0.5}{\mathrm{Ti}}_{0.5}O_{2}$ (110).

All surface models consist of 6 layers without adlayers or 8 layers with a transition metal dioxide monolayer as top and bottom layer, respectively. The most stable rutile surface (110) has two different metal sites in the topmost layer, 5‐fold (5c) and 6‐fold coordinated (6c), compare Figure 1. Various possible M/Ti configurations with M= Nb, Sc, Y within the symmetry restriction described below were calculated for the (110) surface of $M_{x}{\mathrm{Ti}}_{1-x}O_{2}$ with x= 0.5, 1/3, compare Figures S1–S2 in Supporting Information (SI). The experimental sheet conductivity of mesoporous ${\mathrm{Ta}}_{1/3}{\mathrm{Ti}}_{2/3}O_{2}$ was below the required conductivity minimum.^[53]^ Thus, only $M_{0.5}{\mathrm{Ti}}_{0.5}O_{2}$ was calculated for M= Ta. For M=V and Ir, positive segregation energies were predicted.^[47]^ Therefore, $M_{0.5}{\mathrm{Ti}}_{0.5}O_{2}$ with M=V, Ir were only calculated for comparison and x=1/3 was not considered.

The most stable configurations are shown in Table 1. Similar to our previous work,^[22]^ symmetry restrictions were applied to our surface models to avoid artificial, unphysical^[54]^ dipole moments. The dipole correction implemented in VASP was found to not be sufficiently effective for the use of non‐symmetrical surface models in previous work.^[22]^ As a result, the number of cation configurations was reduced to symmetric ones which has to be considered as a simplification.

**Table 1:** Thermodynamically most stable metal cation configurations of doped rutile (110) surfaces with and without adlayers. The 5c‐ and 6c‐metal centers of the topmost layer (without adlayers) are indicated.

$M_{x}{\mathrm{Ti}}_{1-x}O_{2}$	Most stable configuration
${\mathrm{Nb}}_{0.5}{\mathrm{Ti}}_{0.5}O_{2}$	5c‐Ti, 6c‐Nb
${\mathrm{Nb}}_{1/3}{\mathrm{Ti}}_{2/3}O_{2}$	5c‐Ti, 6c‐Nb
${\mathrm{Ta}}_{0.5}{\mathrm{Ti}}_{0.5}O_{2}$	5c‐Ti, 6c‐Ta
$V_{0.5}{\mathrm{Ti}}_{0.5}O_{2}$	5c‐V, 6c‐Ti
${\mathrm{Ir}}_{0.5}{\mathrm{Ti}}_{0.5}O_{2}$	5c‐Ti, 6c‐Ti
${\mathrm{Sc}}_{0.5}{\mathrm{Ti}}_{0.5}O_{2}$	5c‐Sc, 6c‐Ti
${\mathrm{Sc}}_{1/3}{\mathrm{Ti}}_{2/3}O_{2}$	5c‐Ti, 6c‐Ti
$Y_{0.5}{\mathrm{Ti}}_{0.5}O_{2}$	5c‐Y, 6c‐Ti
$Y_{1/3}{\mathrm{Ti}}_{2/3}O_{2}$	5c‐Ti, 6c‐Ti
${\mathrm{RuO}}_{2}$ :${\mathrm{Nb}}_{1/3}{\mathrm{Ti}}_{2/3}O_{2}$	5c‐Ti, 6c‐Nb
${\mathrm{RhO}}_{2}:{\mathrm{Nb}}_{1/3}{\mathrm{Ti}}_{2/3}O_{2}$	5c‐Ti, 6c‐Nb
${\mathrm{TaO}}_{2}:{\mathrm{Nb}}_{1/3}{\mathrm{Ti}}_{2/3}O_{2}$	5c‐Ti, 6c‐Ti
${\mathrm{Nb}}_{1/3}{\mathrm{Ir}}_{1/12}{\mathrm{Ti}}_{7/12}O_{2}$	5c‐Ti/Nb, 6c‐Ti/Nb

Among the investigated models with 50 % Nb or Ta substitution the most stable transition metal configuration is the one with titanium atoms in the upper layers as 5c‐Ti, compare Figure 1 and Table 1. More precisely, the most stable surface of ${\mathrm{Nb}}_{0.5}{\mathrm{Ti}}_{0.5}O_{2}$ has Ti as 5‐fold coordinated metal atom in the topmost layer, and the second layer exhibits either columns of titanium atoms or a symmetric distribution with alternating Ti and Nb atoms, compare Figure S1 in Supporting Information and Table 1. When substituting 33 % of Ti with Nb, the topmost layer consists of 5c‐Ti and 6c‐Nb, and the distribution of the other Nb atoms has no effect on the stability. For ${\mathrm{Ir}}_{0.5}{\mathrm{Ti}}_{0.5}O_{2}$ , the most stable surface comprises exclusively Ti atoms in the top layer (5c‐Ti, 6c‐Ti).

Adding an MO_2_ monolayer on a supporting oxide surface is favorable if both compounds have the same crystal structure, as shown in other theoretical work with rutile(110).^[55]^ There RuO_2_(110) is shown to be stable on TiO_2_(110). Based on this, similar models were considered in this work, adding one monolayer of RuO_2_, RhO_2_ or TaO_2_ on ${\mathrm{Nb}}_{1/3}{\mathrm{Ti}}_{2/3}O_{2}$ , see Table 1. When adding a monolayer of ${\mathrm{RuO}}_{2}$ or ${\mathrm{RhO}}_{2}$ on 33 % Nb substituted rutile, the most stable configuration is the same as without ${\mathrm{MO}}_{2}$ monolayer. However, when the monolayer consists of ${\mathrm{TaO}}_{2}$ , the surface only containing Ti atoms in the outermost layers is most stable. The result of Nb being incorporated in the bulk and Ru agglomerating at the surface is consistent with previous studies. Not only did they predict positive segregation energies for Ru and negative for Nb via theoretical means but also, experimentally, the noble metal was found to segregate from the surface while Nb was incorporated into the ${\mathrm{TiO}}_{2}$ lattice.^[47]^ Within the ${\mathrm{TiO}}_{2}$ lattice, a higher Nb concentration was experimentally found near the surface. This effect was especially pronounced for a Nb concentration of 35 mol%. A Nb concentration of 35 mol% also corresponds to the highest experimentally observed electrical sheet conductivity which is larger than pure ${\mathrm{TiO}}_{2}$ by three orders of magnitude, in agreement with theoretical calculations. Even though the DFT bulk calculations do not consider porosity, the results both in conductivity and phase stability are consistent with experimentally observed trends.^[48]^ At variance, for $M_{0.5}{\mathrm{Ti}}_{0.5}O_{2}$ with M=V , Sc, and Y, M is more stable on the 5c‐position. When the substitution degree is decreased to 33 %, Ti is in the topmost layer in the most stable configuration for $M_{1/3}{\mathrm{Ti}}_{2/3}O_{2}$ with M=Sc, Y.

The *p*‐type doping of rutile with Sc and Y leads to spontaneous oxygen loss, leading to oxygen vacancies and highly reconstructed surfaces as shown previously,^[22]^ thus ${\mathrm{Sc}}_{x}{\mathrm{Ti}}_{1-x}O_{2}$ and $Y_{x}{\mathrm{Ti}}_{1-x}O_{2}$ surfaces are not considered any further.

When substituting Ti atoms with two other transition metals simultaneously, namely Nb and Ir, the most stable configuration contains only Ti and Nb in the topmost layer, see Table 1 and Figure S3 in SI. A cation configuration with Ir at the surface is 0.6 eV less stable.

## Adsorption

**Figure 2:**
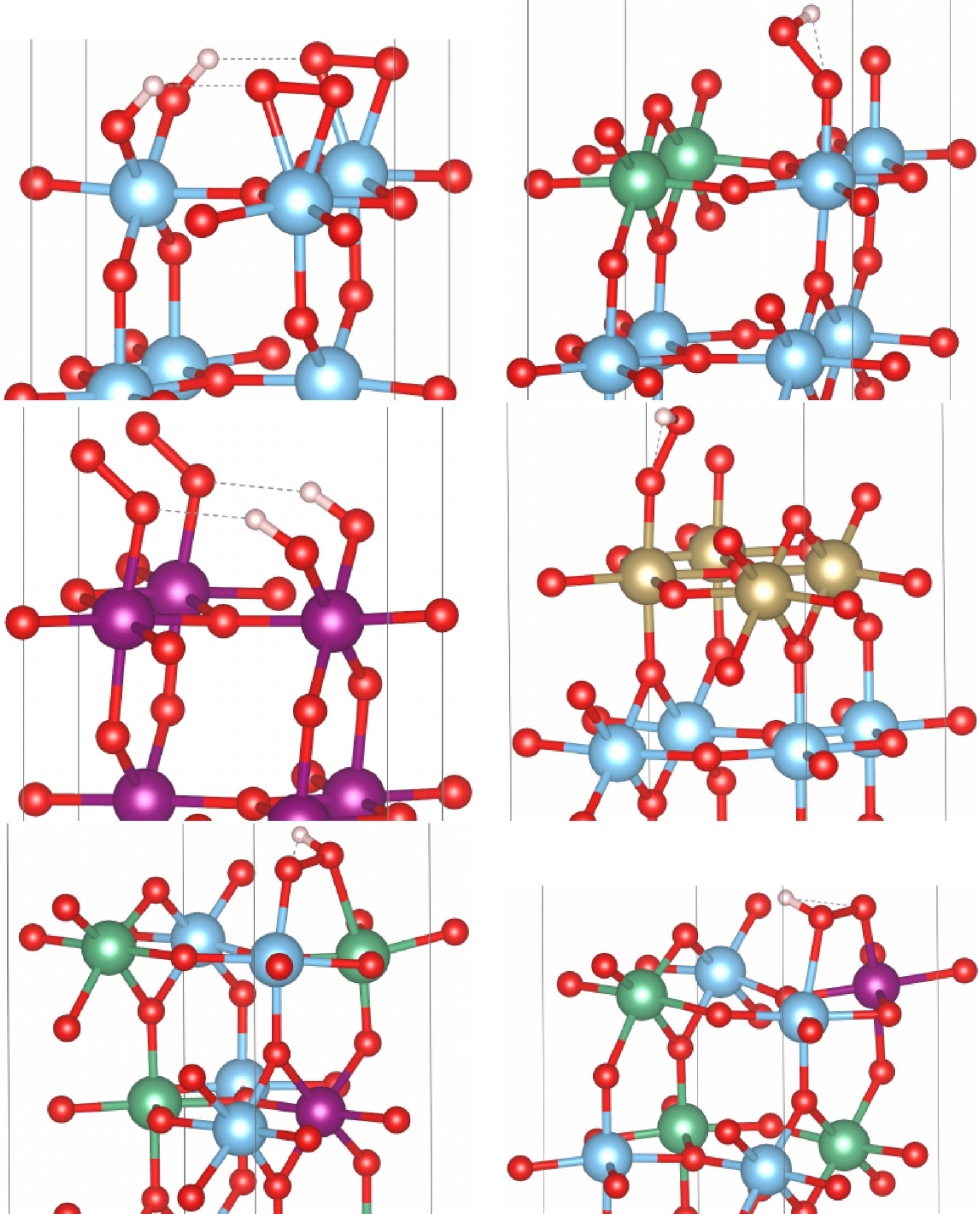
Optimized adsorption structures of M−OOH on ${\mathrm{TiO}}_{2}$ , ${\mathrm{Nb}}_{0.5}{\mathrm{Ti}}_{0.5}O_{2}$ , ${\mathrm{IrO}}_{2}:{\mathrm{Ir}}_{1/3}{\mathrm{Ti}}_{2/3}O_{2}$ , ${\mathrm{TaO}}_{2}$ :${\mathrm{Nb}}_{1/3}{\mathrm{Ti}}_{2/3}O_{2}$ , ${\mathrm{Nb}}_{1/3}{\mathrm{Ir}}_{1/12}{\mathrm{Ti}}_{7/12}O_{2}$ with 5c‐Ti/5c‐Nb and 5c‐Ti/5c‐Ir; Ti: blue spheres, Nb: green spheres, Ir: violet spheres, Ta: gold spheres and O: red spheres; visualized with VESTA.[56]

Adsorption energies of M−O, M−OH and M−OOH were calculated according to the equations from García‐Mota et al.^[32]^ in the conventional AEM/mononuclear mechanism with $H_{2}O$ and $H_{2}$ to avoid the difficult triplet state of molecular oxygen,[^5^, ^37^, ^38^] compare Eq. 19. Only the most stable cation configurations and adsorption modes were taken into account. M−OH and M−OOH are tilted above the metal center. Figure 2 shows the most stable M−OOH adsorbate structures. The optimized structures of ${\mathrm{Nb}}_{0.5}{\mathrm{Ti}}_{0.5}O_{2}$ , ${\mathrm{Nb}}_{1/3}{\mathrm{Ti}}_{2/3}O_{2}$ , ${\mathrm{RuO}}_{2}$ , ${\mathrm{RuO}}_{2}:{\mathrm{TiO}}_{2}$ (monolayers of ${\mathrm{RuO}}_{2}$ supported by ${\mathrm{TiO}}_{2}$ ), ${\mathrm{TaO}}_{2}:{\mathrm{Nb}}_{1/3}{\mathrm{Ti}}_{2/3}O_{2}$ , and ${\mathrm{Nb}}_{1/3}{\mathrm{Ir}}_{1/12}{\mathrm{Ti}}_{7/12}O_{2}$ yield an undissociated M−OOH structure. In contrast, a dissociation into M−OO and H binding to a different surface oxygen atom ${O^{*}}_{A}$ was observed for all other investigated systems. The existence of the M-OOH structure can indicate the AEM/mononuclear[^30^, ^31^] or bifunctional II mechanism^[31]^ whereas the absence of it suggests the bifunctional I[^31^, ^36^] or dissociative/electrochemical oxide pathway.[^34^, ^35^] In previous theoretical work,^[44]^ M−OOH adsorption also partially resulted in M−OO and $H-O_{A}^{*}$ formation.

Spin polarization is relevant for the case of M−O adsorption, but not for M−OH or M−OOH. Consequently, only M−O adsorption and all antiferromagnetic ${\mathrm{RuO}}_{2}$ calculations were spin‐polarized. The adsorption energies of the oxygen species are presented in Table 2. Results for pure rutile ${\mathrm{TiO}}_{2}$ are in good agreement with the work of García‐Mota et al.,^[32]^ regardless of the used dispersion correction, D3(BJ)[^57^, ^58^] or D4.[^50^, ^51^, ^52^] Due to the small differences between the two dispersion corrections, only D3(BJ) was applied for the other systems.

**Table 2:** Adsorption energies calculated for rutile(110) surfaces with and without metal oxide adlayers in eV. PBE−D3(BJ), 900 eV cutoff with exception of ${\mathrm{TiO}}_{2}$ and the structures of ${\mathrm{IrO}}_{2}$ with 450 eV. All calculations are restricted Kohn‐Sham, except for the spin‐polarized cases with M−O and for antiferromagnetic ${\mathrm{RuO}}_{2}$ .

		${\Delta E}_{M-O}$	${\Delta E}_{M-\mathrm{OH}}$	${\Delta E}_{M-\mathrm{OOH}}$	PDS	$\eta^{\mathrm{OER}}$ /V
${\mathrm{TiO}}_{2}$		4.69	1.87	4.13	OH	1.23
‐PBE−D4		4.71	1.87	4.15	OH	1.22
${\mathrm{Nb}}_{0.5}{\mathrm{Ti}}_{0.5}O_{2}$		0.81	−0.71	2.79	OOH	1.42
${\mathrm{Nb}}_{1/3}{\mathrm{Ti}}_{2/3}O_{2}$		1.27	−0.51	2.99	OOH	1.15
${\mathrm{RuO}}_{2}$		1.77	0.20	3.24	OOH	0.87/1.04 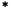
‐ AFM		1.78	0.19	3.23	OOH	0.86/1.03 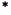
${\mathrm{RuO}}_{2}$ :${\mathrm{TiO}}_{2}$		1.58	0.07	2.87	OOH	0.70
${\mathrm{RuO}}_{2}$ :${\mathrm{Nb}}_{0.5}{\mathrm{Ti}}_{0.5}O_{2}$		1.27	−0.03	2.67	OOH	0.97
${\mathrm{RuO}}_{2}$ :${\mathrm{Nb}}_{1/3}{\mathrm{Ti}}_{2/3}O_{2}$		1.25	−0.03	2.67	OOH	0.99
${\mathrm{IrO}}_{2}$		1.41	−0.45	2.38	$O_{2}$ /OOH	0.57
${\mathrm{IrO}}_{2}$ :${\mathrm{TiO}}_{2}$		1.46	−0.34	2.36	$O_{2}$ (OOH)	0.60
${\mathrm{IrO}}_{2}$ :${\mathrm{Nb}}_{0.5}{\mathrm{Ti}}_{0.5}O_{2}$		1.49	−0.28	2.47	OOH	0.56
${\mathrm{IrO}}_{2}$ :${\mathrm{Nb}}_{1/3}{\mathrm{Ti}}_{2/3}O_{2}$		1.52	−0.22	2.52	OOH	0.58
${\mathrm{IrO}}_{2}$ :${\mathrm{Ir}}_{1/3}{\mathrm{Ti}}_{2/3}O_{2}$		1.31	−0.52	2.80	OOH	1.07
${\mathrm{Ir}}_{0.5}{\mathrm{Ti}}_{0.5}O_{2}$		3.55	0.56	3.79	O	1.14
${\mathrm{RhO}}_{2}$		2.61	0.46	2.68	O	0.31
${\mathrm{RhO}}_{2}$ :${\mathrm{Nb}}_{1/3}{\mathrm{Ti}}_{2/3}O_{2}$		2.27	0.42	2.65	$O_{2}$	0.31
‐ ϵ=78.4 (water)		2.34	0.47	2.72	$O_{2}$	0.23
‐ ϵ=24.5 (ethanol)		2.33	0.46	2.71	$O_{2}$	0.24
${\mathrm{TaO}}_{2}$ :${\mathrm{Nb}}_{1/3}{\mathrm{Ti}}_{2/3}O_{2}$		−0.68	−1.33	2.25	OOH	2.37
${\mathrm{Nb}}_{1/3}{\mathrm{Ir}}_{1/12}{\mathrm{Ti}}_{7/12}O_{2}$	5c‐Ti	0.44	−0.52	2.50	OOH	1.50
	5c‐Nb	−0.30	−0.85	2.43	OOH	2.16
	5c‐Ir	1.52	−0.37	2.43	$O_{2}$	0.72

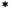
 The overpotential of ${\mathrm{RuO}}_{2}$ is calculated with ZPE, $H_{\mathrm{vib}}$ , TS of ${\mathrm{RuO}}_{2}$ :${\mathrm{TiO}}_{2}$ and ${\mathrm{RuO}}_{2}$ :${\mathrm{Nb}}_{1/3}{\mathrm{Ti}}_{2/3}O_{2}$ , respectively.

**Figure 3:**
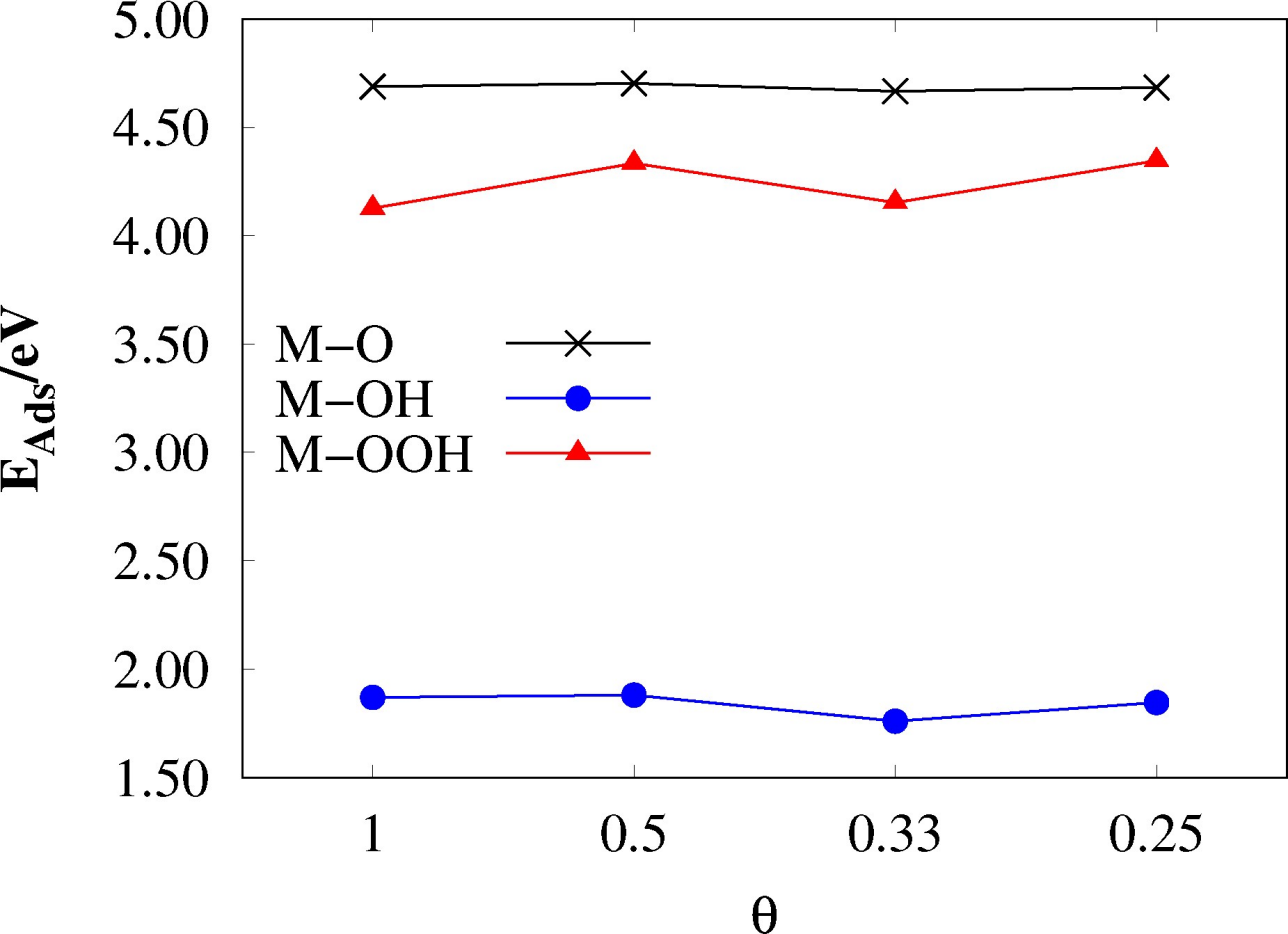
Adsorption energy as a function of surface coverage θ on ${\mathrm{TiO}}_{2}$ (110).

Most adsorption calculations were performed for the primitive surface unit cell (PUC). In order to check whether the PUC is large enough to prevent the adsorbed species from interfering with each other size convergence tests were performed. The adsorption energies calculated with systematically increased surface supercells are shown in Figure 3. The surface coverage θ is the reciprocal number of coordinately unsaturated surface sites, compare Eq. 22. θ=1 corresponds to the PUC, θ=0.5 to a 2×2 , θ=0.33 to a 3×1 and θ=0.25 to a 2×2 supercell.

(22) $$\theta =\frac{\mathrm{number}\;\mathrm{of}\;\mathrm{occupied}\;\mathrm{adsorption}\;\mathrm{sites}}{\mathrm{number}\;\mathrm{of}\;\mathrm{existing}\;\mathrm{adsorption}\;\mathrm{sites}}$$

From Figure 3 it is apparent that only the adsorption energy of the largest adsorbate in this study, M−OOH, is fluctuating slightly with decreasing θ . However, the calculated adsorption energies are already converged within 0.02 eV for M−O, 0.1 eV for M−OH and 0.2 eV for M−OOH for the PUC. In order to reduce computational cost, no supercell construction was performed for the doped structures and adlayers.

When substituting Ti with Nb, all adsorption energies decrease, for both x=1/3 and x=0.5 , compare Table 2. This is most pronounced for M−O and even results in negative adsorption energies for M−OH. The calculated adsorption energies are similar to ${\mathrm{NbTi}}_{15}O_{32}$ .^[32]^ A substitution with 33 % Nb yields larger adsorption energies than with 50 % and closer results compared to pure ${\mathrm{TiO}}_{2}$ .

For models with a ${\mathrm{RuO}}_{2}$ monolayer on ${\mathrm{Nb}}_{1/3}{\mathrm{Ti}}_{2/3}O_{2}$ and ${\mathrm{Nb}}_{0.5}{\mathrm{Ti}}_{0.5}O_{2}$ , similar adsorption energies are obtained, 1.25 eV/1.27 eV for M−O, −0.03 eV for M−OH and 2.67 eV for M−OOH. However, a ${\mathrm{RuO}}_{2}$ monolayer on pure ${\mathrm{TiO}}_{2}$ results in larger adsorption energies of 1.58 eV for M−O, 0.07 eV for M−OH and 2.87 eV for M−OOH. Compared to ${\mathrm{RuTi}}_{15}O_{32}$ (5c‐Ru CUS(Ru)),^[32]^ the adsorption energies of M−O and M−OOH are smaller, in contrast to our work where M−OH has a significantly lower energy.

Pure ${\mathrm{RuO}}_{2}$ yields higher adsorption energies than ${\mathrm{IrO}}_{2}$ but lower than ${\mathrm{TiO}}_{2}$ . All materials with ${\mathrm{IrO}}_{2}$ monolayers and pure ${\mathrm{IrO}}_{2}$ provide negative adsorption energies for M−OH. In comparison to ${\mathrm{IrTi}}_{15}O_{32}$ (5c‐Ir CUS(Ir)),^[32]^ the adsorption of M−O is similar for pure ${\mathrm{IrO}}_{2}$ , ${\mathrm{IrO}}_{2}$ :${\mathrm{TiO}}_{2}$ and ${\mathrm{IrO}}_{2}$ on doped rutile in contrast to the other two Ir‐based materials. For M−OOH, all adsorption energies are higher in this work. The adsorption energies of ${\mathrm{Ir}}_{0.5}{\mathrm{Ti}}_{0.5}O_{2}$ are between the ones of pure ${\mathrm{TiO}}_{2}$ and pure ${\mathrm{IrO}}_{2}$ , as expected. The main difference between ${\mathrm{RhO}}_{2}$ and ${\mathrm{RhO}}_{2}$ :${\mathrm{Nb}}_{1/3}{\mathrm{Ti}}_{2/3}O_{2}$ is the adsorption energy of oxygen, the other adsorption energies are similar.

The OER takes place at a surface adjacent to an electrolyte. The explicit theoretical treatment of a solid/liquid interface, which is desirable, is highly demanding due to the large number of particles, many degrees of freedom, and long simulation times in molecular dynamics simulations. In the present study we therefore used an implicit solvation model as an approximation by applying the software package VASPsol.[^59^, ^60^, ^61^] Two permittivity values were used, corresponding to water^[62]^ and ethanol,^[63]^ exemplarily for the ${\mathrm{RhO}}_{2}$ :${\mathrm{Nb}}_{1/3}{\mathrm{Ti}}_{2/3}O_{2}$ surface model, compare Table 2. Even though the use of the implicit solvation model led to slightly lower overpotentials, the differences with respect to the vacuum calculations are minor and were therefore omitted for the other systems to save computational time.

${\mathrm{TaO}}_{2}$ :${\mathrm{Nb}}_{1/3}{\mathrm{Ti}}_{2/3}O_{2}$ yields the highest overpotential in this work due to its negative adsorption energies for M−O and the large difference to M−OOH. For the adsorption on the ternary oxide ${\mathrm{Nb}}_{1/3}{\mathrm{Ir}}_{1/12}{\mathrm{Ti}}_{7/12}O_{2}$ , various adsorption sites were tested, namely 5c‐Ti, 5c‐Nb and 5c‐Ir. Depending on the metal atom on which the adsorption takes place, there are considerable differences in adsorption energies resulting in different catalytic activities. Although the formation of M−OOH yields similar energies and the energies of M−OH are all negative, the oxygen adsorption differs strongly depending on the CUS metal. When Nb is the corresponding metal, the oxygen as well as the M−OH adsorption energy are negative, similar to ${\mathrm{TaO}}_{2}:{\mathrm{Nb}}_{1/3}{\mathrm{Ti}}_{2/3}O_{2}$ . For 5c‐Ti, the adsorption energies resemble the ones of 50 % Nb‐doped rutile. When the adsorption takes place on iridium, the adsorption energies are similar to pure or adlayer ${\mathrm{IrO}}_{2}$ .

## Frequency Calculations

For calculating the Gibbs free energy ΔG according to Eq. 23, frequency calculations were performed for $H_{2}$ , $H_{2}O$ and adsorption intermediates on the surface of selected structures.

(23) $$\Delta G=\Delta E+\Delta \mathrm{ZPE}+H_{\mathrm{vib}}-T\Delta S_{\mathrm{vib}}$$

The resulting zero point energies (ZPE), enthalpy $H_{\mathrm{vib}}$ , and entropic (TS) corrections at T= 298 K are shown in Table 3 and 4. ZPE, $H_{\mathrm{vib}}$ and TS of molecular hydrogen reproduce the literature values well.[^5^, ^32^, ^33^] ZPE and $H_{\mathrm{vib}}$ of water are also similar to literature.[^32^, ^33^] However, its entropic term is too low. For the reaction Gibbs Energies $\Delta G^{\mathrm{OER}}$ , literature values for $H_{2}$ ^[5]^ and $H_{2}O$ ^[32]^ were used. During the frequency calculations of the surfaces, the inner layers were fixed except for pure ${\mathrm{TiO}}_{2}$ . Thus, the absolute values of ZPE, $H_{\mathrm{vib}}$ and TS are not comparable, only their differences. For the vibrational corrections of the adsorption intermediates, the values for M−O are between −0.1 and 0.1 eV, for M−OH about 0.5 eV for most structures and for M−OOH between 0.5 and 0.7 eV, compare Table 4. When employing ${\mathrm{RuO}}_{2}$ as an adlayer, the vibrational corrections are similar for 50 % and 33 % Nb‐substitution. In particular for M−O, the ZPE$+H_{\mathrm{vib}}-$ TS are closer if the supporting material is the same. Compared to García‐Mota et al.,^[32]^ the individual vibrational corrections are much higher, but their sums are in the same range.

**Table 3:** Calculated vibrational corrections (ZPE+$H_{\mathrm{vib}}$ ) and entropic corrections (TS) with T=298 K in comparison with literature values of $H_{2}$ and $H_{2}O$ in eV.

	ZPE+$H_{\mathrm{vib}}$	TS
$H_{2}\left(g\right)$	0.27	0.45
Literature 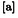	0.27	0.40
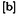	0.27	0.41
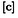	0.35	0.40
$H_{2}O$	0.57	0.29
Literature 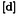	0.58 (1 bar)	0.67 (0.035 bar)
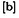	0.56	0.67 (0.035 bar)
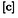	0.57	0.67 (0.035 bar)

[a]^[5]^ [b]^[33]^ [c]^[32]^ [d]^[64]^

**Table 4:** Calculated vibrational corrections (ZPE+$H_{\mathrm{vib}}$ ) and entropic corrections (TS) with T=298 K and their difference Δ of the adsorption intermediates in eV.

		ZPE+$H_{\mathrm{vib}}$	TS	Δ
${\mathrm{TiO}}_{2}$	−O	2.31	2.27	0.04
	−OH	2.96	2.40	0.55
	−OOH	3.21	2.48	0.74
${\mathrm{Nb}}_{1/3}{\mathrm{Ti}}_{2/3}O_{2}$	−O	0.77	0.86	−0.09
	−OH	1.33	0.87	0.47
	−OOH	1.50	0.96	0.54
${\mathrm{IrO}}_{2}$ :${\mathrm{Nb}}_{1/3}{\mathrm{Ti}}_{2/3}O_{2}$	−O	0.81	0.84	−0.04
	−OH	1.40	0.88	0.52
	−OOH	1.63	0.90	0.73
${\mathrm{RhO}}_{2}$ :${\mathrm{Nb}}_{1/3}{\mathrm{Ti}}_{2/3}O_{2}$	−O	0.78	0.84	−0.06
	−OH	1.36	0.88	0.48
	−OOH	1.63	0.90	0.73
${\mathrm{RuO}}_{2}$ :${\mathrm{TiO}}_{2}$	−O	0.84	0.73	0.11
	−OH	1.42	0.78	0.64
	−OOH	1.54	0.83	0.71
${\mathrm{RuO}}_{2}$ :${\mathrm{Nb}}_{0.5}{\mathrm{Ti}}_{0.5}O_{2}$	−O	0.75	0.85	−0.10
	−OH	1.32	0.89	0.44
	−OOH	1.57	0.90	0.67
${\mathrm{RuO}}_{2}$ :${\mathrm{Nb}}_{1/3}{\mathrm{Ti}}_{2/3}O_{2}$	−O	0.75	0.85	−0.10
	−OH	1.33	0.88	0.44
	−OOH	1.57	0.90	0.67

Adopting the approach by García‐Mota et al.,^[32]^ ZPE, $H_{\mathrm{vib}}$ and TS of similar models were employed to save computational time. For this reason, the corrections for the Gibbs energy of ${\mathrm{Nb}}_{1/3}{\mathrm{Ti}}_{2/3}O_{2}$ were also used for ${\mathrm{Nb}}_{0.5}{\mathrm{Ti}}_{0.5}O_{2}$ and ${\mathrm{Nb}}_{1/3}{\mathrm{Ir}}_{1/12}{\mathrm{Ti}}_{7/12}O_{2}$ , for pure ${\mathrm{RhO}}_{2}$ the ZPE, $H_{\mathrm{vib}}$ and TS of ${\mathrm{RhO}}_{2}$ :${\mathrm{Nb}}_{1/3}{\mathrm{Ti}}_{2/3}O_{2}$ , the ones of ${\mathrm{IrO}}_{2}$ :${\mathrm{Nb}}_{1/3}{\mathrm{Ti}}_{2/3}O_{2}$ for all Ir‐based structures. García‐Mota et al.^[32]^ calculated ZPE for the adsorbed species on pure ${\mathrm{TiO}}_{2}$ and considered it for the other doped rutile structures ${\mathrm{MTi}}_{15}O_{32}$ . Furthermore, they considered the entropic corrections to be zero due to the translational entropy considered being the main contribution.^[32]^

## Reaction Diagrams and Overpotential

**Figure 4:**
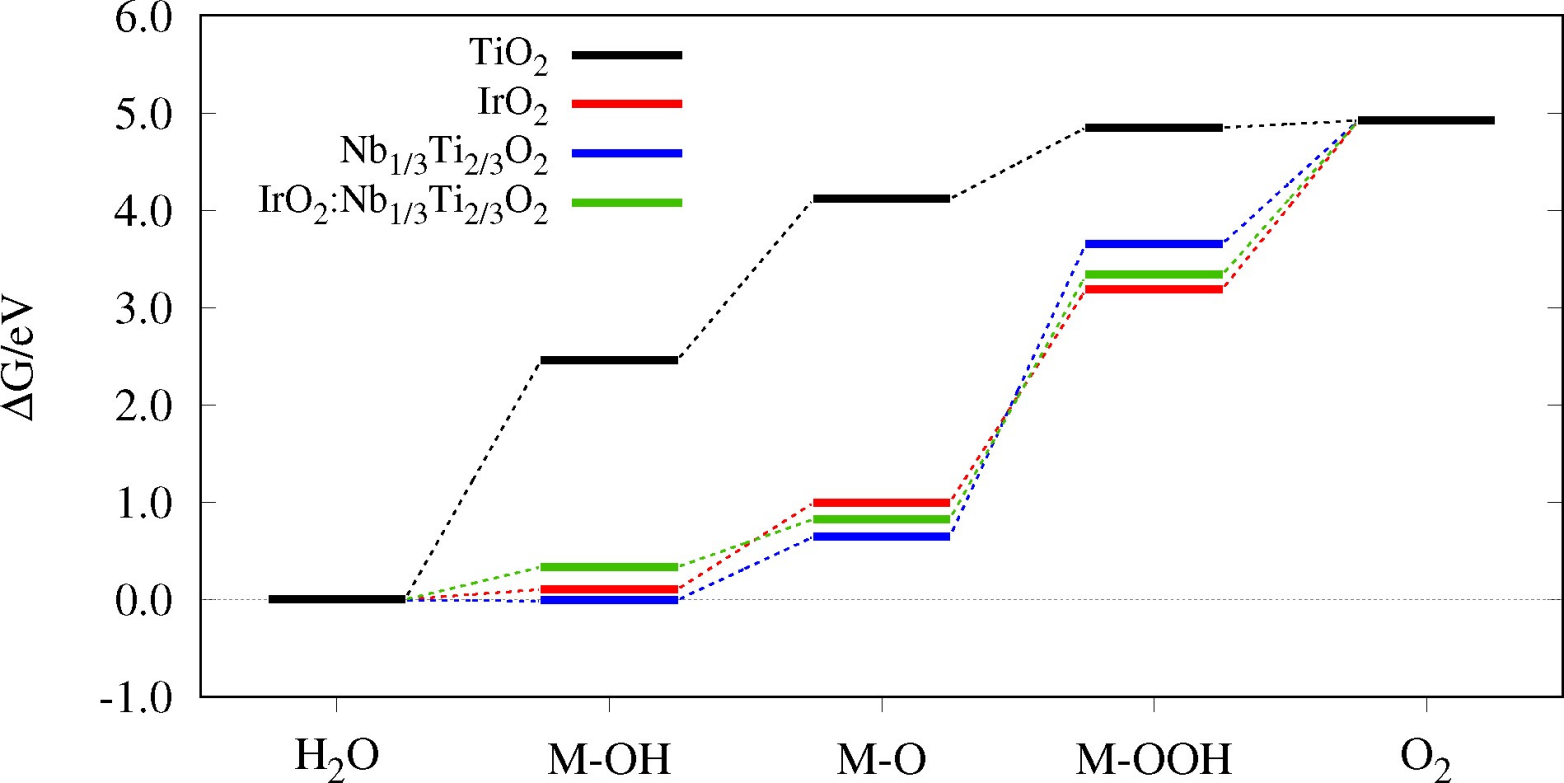
Reaction diagram of ${\mathrm{TiO}}_{2}$ , ${\mathrm{IrO}}_{2}$ , ${\mathrm{Nb}}_{1/3}{\mathrm{Ti}}_{2/3}O_{2}$ and ${\mathrm{IrO}}_{2}$ :${\mathrm{Nb}}_{1/3}{\mathrm{Ti}}_{2/3}O_{2}$ .

**Figure 5:**
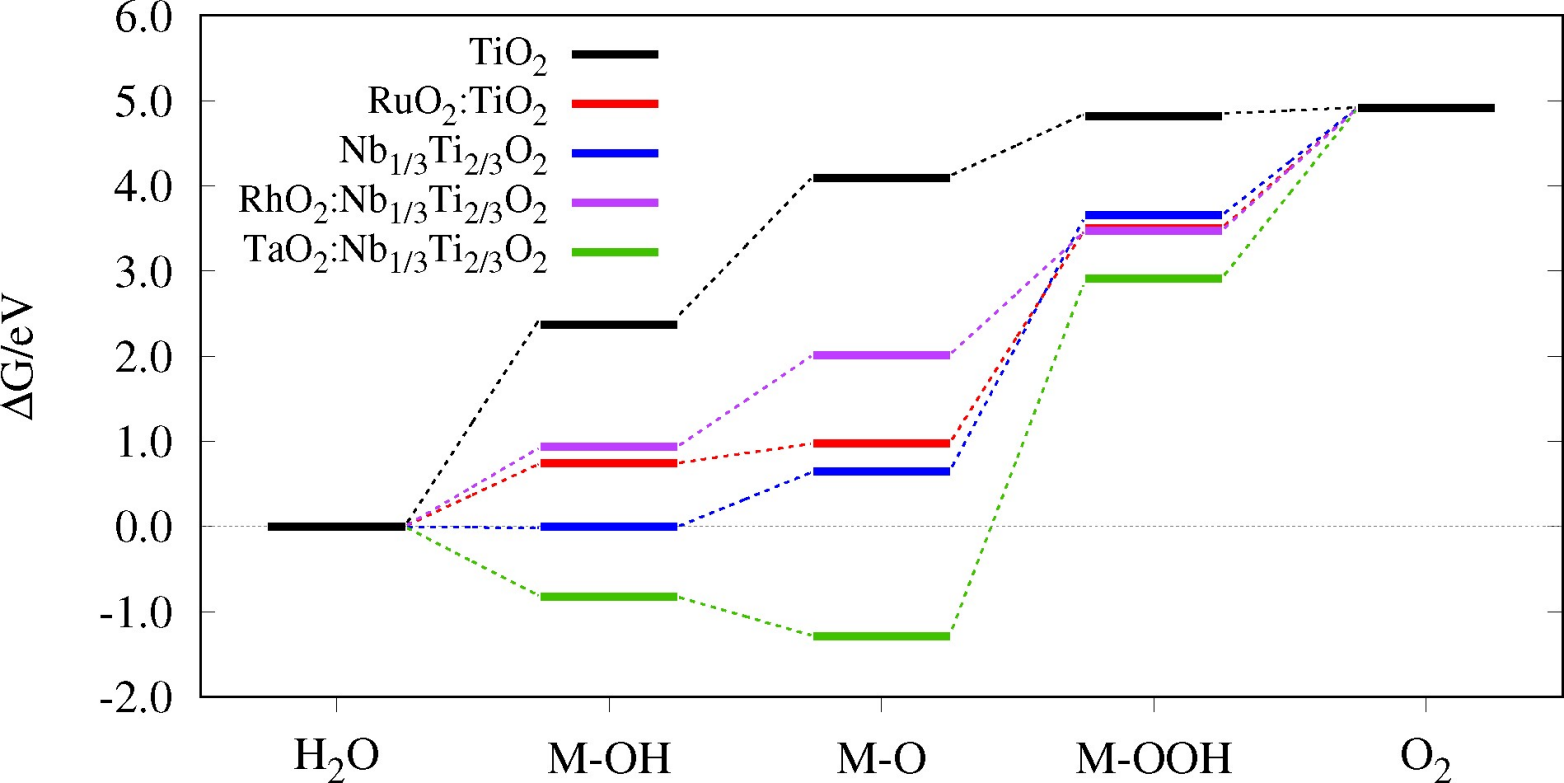
Reaction diagram of the structures with adlayers in comparison to their supporting material.

**Figure 6:**
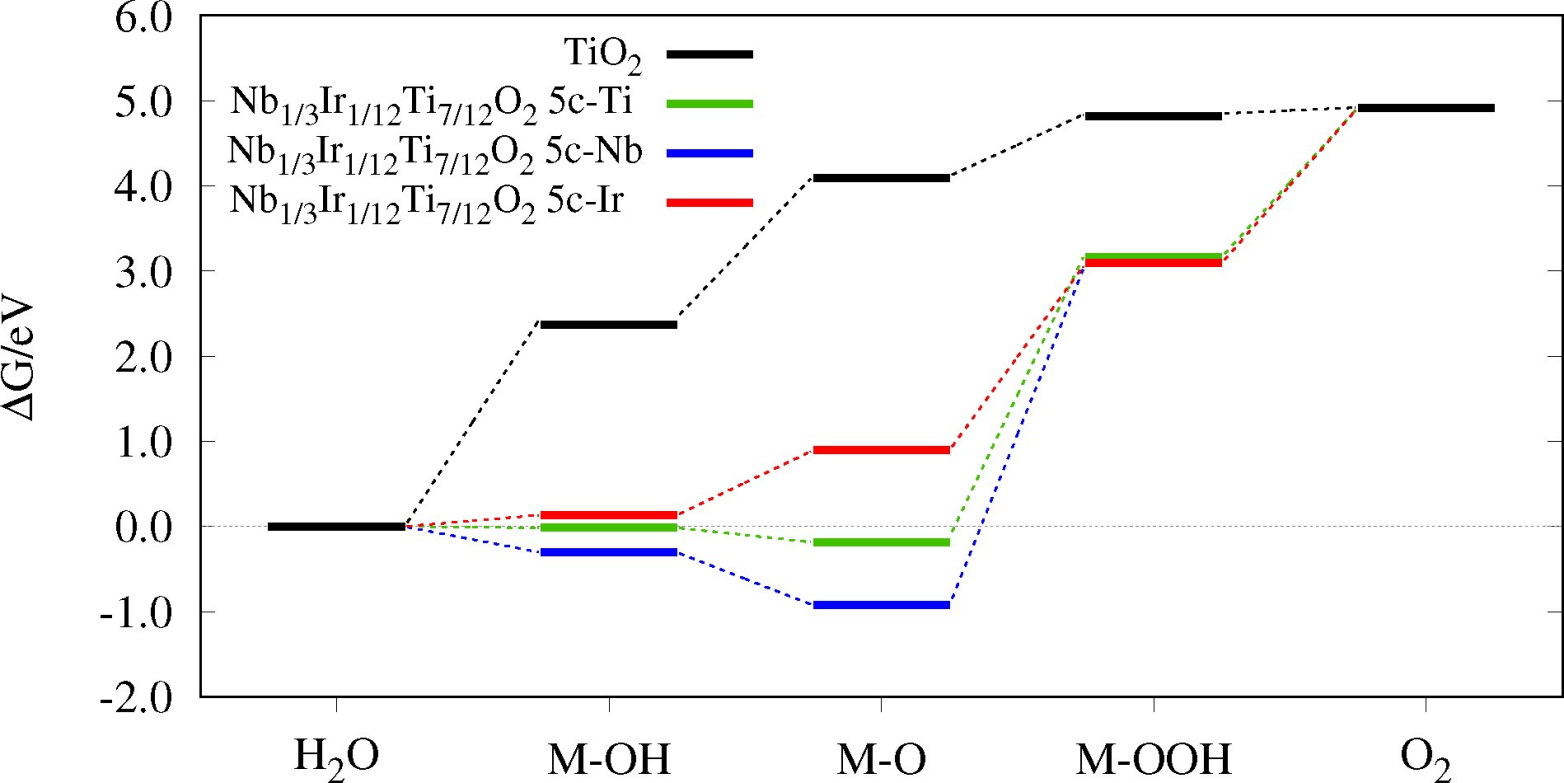
Reaction diagram of ${\mathrm{Nb}}_{1/3}{\mathrm{Ir}}_{1/12}{\mathrm{Ti}}_{7/12}O_{2}$ with different adsorption sites (5c‐Ti, 5c‐Nb, 5c‐Ir) in comparison to ${\mathrm{TiO}}_{2}$ .

The potential determining step (PDS) and the overpotential $\eta^{\mathrm{OER}}$ are determined by including calculated vibrational corrections. The overpotential is dependent on the PDS, the largest energy difference in the reaction, so that smaller $\eta^{\mathrm{OER}}$ values predict a better electrocatalytic activity, compare Eq. 21. During a reaction, the reactants change from an initial state via intermediates to a final state along a reaction pathway, specified as the reaction coordinate, see Figures 4–6. After calculating the Gibbs free energies $\Delta G^{\mathrm{OER}}$ of the four AEM reaction steps, the energy differences were computed to obtain the PDS and the overpotential $\eta^{\mathrm{OER}}$ , see Table 2. While absolute values of theoretical and experimental overpotentials are difficult to compare, a comparison of trends and differences is valid.[^36^, ^42^] At electrochemical equilibrium potential with U= 1.23 V vs. reversible hydrogen electrode, theoretical calculations suggest that the AEM/mononuclear mechanism, bifunctional I, and bifunctional II are the most favorable pathways depending on $\Delta G_{1}$ . For the most active catalysts, bifunctional I is the most important one, which has M−OO and $O_{A}^{*}$ as intermediates.^[31]^ Other DFT calculations and microkinetic modeling suggest a competition between M−OOH formation and O−O coupling at the rutile(110) surface of ${\mathrm{IrO}}_{2}$ , ${\mathrm{RuO}}_{2}$ , ${\mathrm{RhO}}_{2}$ , and ${\mathrm{PtO}}_{2}$ .^[40]^ Selected reaction diagrams are shown in Figure 4, 5, 6. The Gibbs free energy of water was set to zero to match the formation energy of $O_{2}$ as the sum of the Gibbs free energies.[^32^, ^33^, ^34^] For molecular oxygen, the value of 4.92 V was taken from experiment.[^32^, ^33^, ^34^] In Figure 4, pure ${\mathrm{TiO}}_{2}$ has different adsorption Gibbs free energies compared to the other systems. The energies of the reaction steps are far from being equal, which should be the case for an ideal OER catalyst. Here, the formation of M−OH is the potential determining step. In other work,^[32]^ the PDS of pure rutile is the formation of M−O. In this work, the PDS, M−OH formation, is slightly more demanding but the Gibbs energies are very similar. The resulting overpotential of 1.23 V is also similar to 1.30 V.^[32]^ Calculations with the D4 dispersion correction[^51^, ^52^] were performed for pure ${\mathrm{TiO}}_{2}$ . Employing the D4 correction[^51^, ^52^] leads to slightly larger adsorption energies in comparison to D3(BJ)[^57^, ^58^] but the descriptor is very similar. In other work, ${\mathrm{TiO}}_{2}$ has an overpotential of 1.19 V, which is very similar to our results, but the formation of M−O is the potential determining step.^[43]^ Other calculations of two different surface terminations of ${\mathrm{TiO}}_{2}$ gave the first step as PDS, the formation of M−OH,^[33]^ as in this work.

In contrast, the state‐of‐the‐art OER catalyst ${\mathrm{IrO}}_{2}$ has reaction energy differences closer to $\Delta G_{O_{2}}^{\mathrm{OER}}/4=1.23\;\mathrm{eV}$ leading to a much smaller overpotential, 0.57 V. The difference between the formation of molecular oxygen and of M−OOH is less than 0.02 eV for pure ${\mathrm{IrO}}_{2}$ and 0.13 eV for ${\mathrm{IrO}}_{2}$ :${\mathrm{TiO}}_{2}$ , so both could be the PDS. When ${\mathrm{IrO}}_{2}$ is only used as an adlayer with another supporting oxide, the overpotentials are still ≈0.6V and thus close to pure ${\mathrm{IrO}}_{2}$ , except for ${\mathrm{IrO}}_{2}$ :${\mathrm{Ir}}_{1/3}{\mathrm{Ti}}_{2/3}O_{2}$ . This is in line with the positive segregation energies of ${\mathrm{Ir}}_{0.5}{\mathrm{Ti}}_{0.5}O_{2}$ and ${\mathrm{Ir}}_{0.25}{\mathrm{Ti}}_{0.75}O_{2}$ calculated theoretically and the experimentally observed surface segregation of Ir.^[47]^ Other work also found that Ir‐doped ${\mathrm{TiO}}_{2}$ is unstable.^[32]^ M−O is too strongly stabilized and leads to the increase of the energy of the PDS, the M−OOH formation. Compared to ${\mathrm{IrTi}}_{15}O_{32}$ (5c‐Ir),^[32]^ the overpotentials are similar and the M−OOH formation is also the PDS. Experimentally, ${\mathrm{IrO}}_{2}$ nanoparticles on ${\mathrm{Nb}}_{0.05}{\mathrm{Ti}}_{0.95}O_{2}$ powders showed improved stability in comparison to unsupported ${\mathrm{IrO}}_{2}$ . When the ${\mathrm{IrO}}_{2}$ ‐loading is below 26 wt% it becomes discontinuous which leads to an interrupted conductive path.^[65]^ In line with the present results, previous theoretical studies have shown the PDS of the oxygen covered ${\mathrm{IrO}}_{2}$ (110) surface is the formation of M−OOH with an overpotential of 0.56 V.^[43]^ Experimentally, all accessible Ir sites on Ir/${\mathrm{TiO}}_{x}$ contribute equally to the OER above 25 wt% Ir.^[27]^

Here, ${\mathrm{Ir}}_{0.5}{\mathrm{Ti}}_{0.5}O_{2}$ has a lower overpotential than pure ${\mathrm{TiO}}_{2}$ , but a higher one than pure ${\mathrm{IrO}}_{2}$ or ${\mathrm{IrO}}_{2}$ on (niobium doped) ${\mathrm{TiO}}_{2}$ . The higher Ir : Ti ratio worsens the descriptor in comparison to ${\mathrm{IrO}}_{2}$ :${\mathrm{Ir}}_{1/3}{\mathrm{Ti}}_{2/3}O_{2}$ , in line with previous results on ${\mathrm{Ir}}_{0.5}{\mathrm{Ti}}_{0.5}O_{2}$ and ${\mathrm{Ir}}_{0.25}{\mathrm{Ti}}_{0.75}O_{2}$ which exhibit positive segregation energies.^[47]^ In contrast to pure ${\mathrm{TiO}}_{2}$ , pure ${\mathrm{IrO}}_{2}$ , or ${\mathrm{IrO}}_{2}$ as an adlayer, the PDS of ${\mathrm{Ir}}_{0.5}{\mathrm{Ti}}_{0.5}O_{2}$ is the formation of M−O.

For the oxygen reduction reaction (ORR), the overpotential on metal surfaces are the smallest for Ir and Rh,^[34]^ which also matches the metal oxide results in this study.

${\mathrm{RuO}}_{2}$ is a better OER catalyst than ${\mathrm{IrO}}_{2}$ in acidic media, but the dissolution is higher.^[66]^ During OER, ${\mathrm{RuO}}_{2}$ corrodes to ${\mathrm{RuO}}_{4}$ .^[67]^ In other work, ${\mathrm{RuO}}_{2}$ has the same PDS yet shows a lower overpotential of 0.37 V.^[43]^ Experimental isotope labeling and differential electrochemical mass spectrometry was used to show the partition of the oxide layer in the oxygen evolution process on ${\mathrm{RuO}}_{2}$ and Ru.^[68]^ In this work, no lattice oxygen participated in the oxygen evolution reaction, which could be the reason why the activity is not as good as expected. Other work suggests the opposite, oxygen evolution reaction on crystalline ${\mathrm{RuO}}_{2}$ without oxygen lattice exchange.^[69]^ Experiments with ${\mathrm{RuO}}_{2}$ and ${\mathrm{Ru}}_{0.9}{\mathrm{Ni}}_{0.1}O_{2-\delta }$ involving isotope labeling show that the participation of lattice oxygen in the reaction mechanism depends on the potential, as it partakes at potentials over 1.12 V.^[70]^ For pure ${\mathrm{RuO}}_{2}$ , operando synchrotron Fourier‐transform infrared spectroscopy suggest a Ru‐OOH intermediate.^[71]^ Our geometry optimizations of M−OOH support this. Other work, including experiments and DFT calculations, rule out lattice oxygen participation, but indicate formation of an OO‐species on single‐crystal ${\mathrm{RuO}}_{2}$ (110), which was stabilized by a neighboring OH‐group.^[72]^ The overpotential of pure ${\mathrm{RuO}}_{2}$ depends on the used entropy correction and zero point energy, see Table 2. When the values of ${\mathrm{RuO}}_{2}$ :${\mathrm{TiO}}_{2}$ are employed, its overpotential lies between ${\mathrm{RuO}}_{2}$ :${\mathrm{TiO}}_{2}$ and ${\mathrm{RuO}}_{2}$ on Nb‐doped rutile. When using the values for ${\mathrm{RuO}}_{2}$ :${\mathrm{Nb}}_{1/3}{\mathrm{Ti}}_{2/3}O_{2}$ , the overpotential is close to the ones of ${\mathrm{RuO}}_{2}$ on niobium‐doped rutile with both percentages of niobium substitution. Independent of the employed thermodynamic corrections, the potential determining step for all materials with ${\mathrm{RuO}}_{2}$ on top of the surface layer is the formation of M−OOH. The overpotential of ${\mathrm{RuTi}}_{15}O_{32}$ ^[32]^ is between 0.58 eV and 0.80 eV for 5c‐ or 6c‐substitution.

By substituting titanium with Nb in ${\mathrm{TiO}}_{2}$ , a decrease in the descriptor from 1.23 to 1.15 V is only detected for ${\mathrm{Nb}}_{1/3}{\mathrm{Ti}}_{2/3}O_{2}$ , but not for ${\mathrm{Nb}}_{0.5}{\mathrm{Ti}}_{0.5}O_{2}$ . The first improves the overpotential, but not to the same extent that was found by García‐Mota et al.^[32]^ The PDS of both niobium substitutions is the formation of M−OOH, which is only the case for the adsorption on Nb for ${\mathrm{NbTi}}_{15}O_{32}$ .^[32]^

When the titanium atoms in pure rutile are substituted by 50 % niobium, the Gibbs energies become more similar to those of ${\mathrm{IrO}}_{2}$ , see Figure 4.

The largest difference between the adsorption energies of ${\mathrm{RhO}}_{2}$ and ${\mathrm{RhO}}_{2}$ :${\mathrm{Nb}}_{1/3}{\mathrm{Ti}}_{2/3}O_{2}$ is the oxygen adsorption, which is also the potential determining step for pure ${\mathrm{RhO}}_{2}$ in contrast to the oxygen molecule formation for ${\mathrm{RhO}}_{2}$ :${\mathrm{Nb}}_{1/3}{\mathrm{Ti}}_{2/3}O_{2}$ . The resulting overpotentials are essentially the same. When employing a solvent model in the surface calculation of ${\mathrm{RhO}}_{2}$ :${\mathrm{Nb}}_{1/3}{\mathrm{Ti}}_{2/3}O_{2}$ , the overpotential is slightly lowered.

For the structures with the lowest overpotential, namely ${\mathrm{RhO}}_{2}$ and ${\mathrm{RhO}}_{2}$ :${\mathrm{Nb}}_{1/3}{\mathrm{Ti}}_{2/3}O_{2}$ , also *first‐principles* molecular dynamics simulations were performed to evaluate the kinetic stability. For these calculations the machine‐learning potential implemented in VASP^[73]^ was employed. In order to reduce computational effort, a lower cutoff energy, 400 eV, was employed. The simulations were performed at 300 and 600 K for 100 ps with a timestep of 1 fs. The structures did not undergo reconstruction, which also indicates kinetic stability.

Consistent with previous studies,^[47]^ Nb is incorporated in the bulk and Rh at the surface. Incorporation of Nb is expected to induce a sufficiently high conductivity for the OER activity.^[48]^

The effect of adlayer addition on the adsorption energies on pure ${\mathrm{TiO}}_{2}$ or ${\mathrm{Nb}}_{1/3}{\mathrm{Ti}}_{2/3}O_{2}$ is presented in Figure 5. Adding a ${\mathrm{RuO}}_{2}$ ‐monolayer to pure ${\mathrm{TiO}}_{2}$ (or Nb doped ${\mathrm{TiO}}_{2}$ in Table 2) strongly decreases the adsorption energies with respect to pure rutile. ${\mathrm{RhO}}_{2}$ on 50 % niobium substituted rutile leads to more balanced adsorption energies. ${\mathrm{TaO}}_{2}$ monolayers, on the contrary, overly stabilize the adsorption of M−OH and M−O, resulting in large energy differences and therefore a larger overpotential with respect to pure ${\mathrm{TiO}}_{2}$ . As Figure 5 and Table 2 underline, it is also the largest overpotential in this study.

The effect of different adsorption sites on the adsorption energies is presented in Figure 6. In ${\mathrm{Nb}}_{1/3}{\mathrm{Ir}}_{1/12}{\mathrm{Ti}}_{7/12}O_{2}$ , all three transition metals can function as the coordinatively unsaturated site. When the adsorption takes place on titanium or niobium, the first two oxygen intermediates are stabilized. For Nb, this leads to negative adsorption energies. In contrast, when the adsorption takes place on 5c‐Ir, the energy differences are much smaller leading to a better overpotential, as expected for Ir. Unfortunately, the most stable surface is not the one with Ir in the topmost layer, but with Ti and Nb. Thus, the structures with better activity are not stable. For the most stable surface, the activity would correspond to the descriptor of 5c‐Ti or 5c‐Nb and the descriptor would not improve or even worsen compared to pure ${\mathrm{TiO}}_{2}$ . If the adsorption took place on Ir, the descriptor would be similar to the other Ir‐based materials. If it were possible to control the configuration of the metal cation experimentally, the overpotential and thus the activity could be tuned. The lowest overpotential of the considered systems is calculated for ${\mathrm{RhO}}_{2}$ and ${\mathrm{RhO}}_{2}$ :${\mathrm{Nb}}_{1/3}{\mathrm{Ti}}_{2/3}O_{2}$ , 0.31 V, 0.31 V and 0.23 V/0.24 V calculated with relative permittivity, respectively, which is even lower than for pure ${\mathrm{IrO}}_{2}$ (0.57 V). Unfortunately, rhodium is also rare^[5]^ and expensive, but still cheaper than iridium.^[74]^

**Figure 7:**
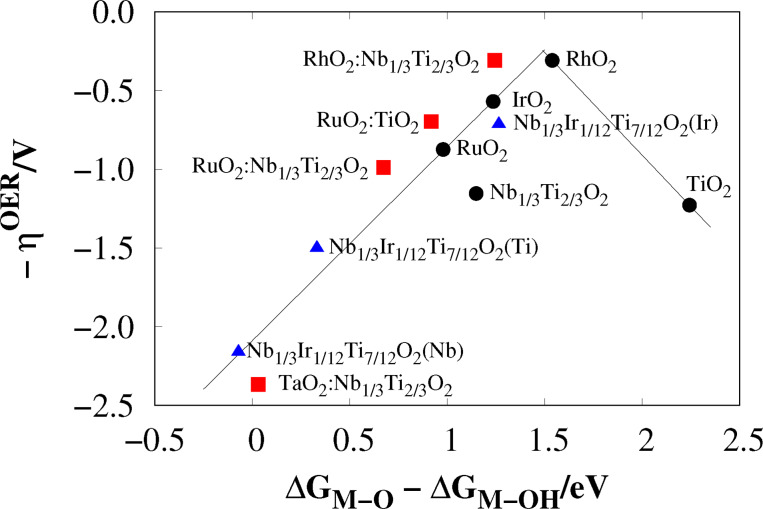
Volcano plot of selected structures. Circle: ${\mathrm{MO}}_{2}$ , square: adlayers, triangle: ${\mathrm{Nb}}_{1/3}{\mathrm{Ir}}_{1/12}{\mathrm{Ti}}_{7/12}O_{2}$ (5c‐M).

Furthermore, in Figure 7 the negative overpotential is plotted against $\Delta G_{M-O}-\Delta G_{M-OH}$ [^32^, ^42^] resulting in a volcano‐type plot. ${\mathrm{RhO}}_{2}$ and ${\mathrm{RhO}}_{2}$ :${\mathrm{Nb}}_{1/3}{\mathrm{Ti}}_{2/3}O_{2}$ are close to the peak. Pure ${\mathrm{TiO}}_{2}$ is the only structure here that is on the right side of this plot, similar to previous work.^[42]^ ${\mathrm{TiO}}_{2}$ binds M−OH too weakly, which results in the M−OH formation as the PDS, compare Table 2. All other structures are on the left side of the maximum. All these structures, except for the iridium‐based ones over‐stabilize M−O, which results in M−OOH formation as the PDS. $O_{2}$ and OOH formation are similarly demanding for ${\mathrm{IrO}}_{2}$ . ${\mathrm{IrO}}_{2}$ is close to the peak with similar $\Delta G_{M-O}-\Delta G_{M-OH}$ values to ${\mathrm{RhO}}_{2}$ :${\mathrm{Nb}}_{1/3}{\mathrm{Ti}}_{2/3}O_{2}$ , the latter has $O_{2}$ formation as the PDS.

## Bader Charge Analysis and Band Structures

In order to further analyze the OER activity, Bader charge analysis was performed and band structures were calculated with wannier90.^[75]^ Bader charges of selected structures are displayed in Table 5. Both Ti and Nb have charges between 2.5 and 2.6e . Ru as an ${\mathrm{RuO}}_{2}$ ‐adlayer has a smaller charge of 2.0 or 1.8e . Similarly, Iridium has a charge of 2.0e . The best catalyst found in this work corresponds to the smallest Bader charge of 1.8 or even 1.6e . The structure with the highest overpotential, with ${\mathrm{TaO}}_{2}$ as an adlayer, also exhibits the highest Bader charge of 4.1e . Bader charges of oxygen all are in the same range of about −1.2e with ${\mathrm{IrO}}_{2}$ and ${\mathrm{RhO}}_{2}$ having the smallest negative charge. For ${\mathrm{Nb}}_{1/3}{\mathrm{Ir}}_{1/12}{\mathrm{Ti}}_{7/12}O_{2}$ , the configuration with iridium at the 5c‐site has a smaller Bader charge of 1.4e for iridium in comparison to 1.7e for 5c‐Ti, 5c‐Nb. It follows that the best OER catalysts also feature the lowest Bader charges.

**Table 5:** Calculated average atomic Bader charges in e .

				${\mathrm{Nb}}_{1/3}{\mathrm{Ir}}_{1/12}{\mathrm{Ti}}_{7/12}O_{2}$		
	Ti	M	*adlayer*‐M	−Nb	−Ir	O
${\mathrm{TiO}}_{2}$	2.6	–	–	–	–	−1.3
${\mathrm{Nb}}_{1/3}{\mathrm{Ti}}_{2/3}O_{2}$	2.5	2.6	–	–	–	−1.3
${\mathrm{RuO}}_{2}$ :${\mathrm{TiO}}_{2}$	2.6	–	2.0	–	–	−1.2
${\mathrm{RuO}}_{2}$ :${\mathrm{Nb}}_{1/3}{\mathrm{Ti}}_{2/3}O_{2}$	2.6	2.6	1.8	–	–	−1.2
${\mathrm{IrO}}_{2}$	–	2.0	–	–	–	−1.0
${\mathrm{RhO}}_{2}$	–	1.8	–	–	–	−0.9
${\mathrm{RhO}}_{2}$ :${\mathrm{Nb}}_{1/3}{\mathrm{Ti}}_{2/3}O_{2}$	2.6	2.6	1.6	–	–	−1.2
${\mathrm{TaO}}_{2}$ :${\mathrm{Nb}}_{1/3}{\mathrm{Ti}}_{2/3}O_{2}$	2.5	2.5	4.1	–	–	−1.4
${\mathrm{Nb}}_{1/3}{\mathrm{Ir}}_{1/12}{\mathrm{Ti}}_{7/12}O_{2}$						
‐5c‐Ti, 5c‐Nb	2.5	–	–	2.6	1.7	−1.2
‐5c‐Ir	2.5	–	–	2.6	1.4	−1.2

**Figure 8:**
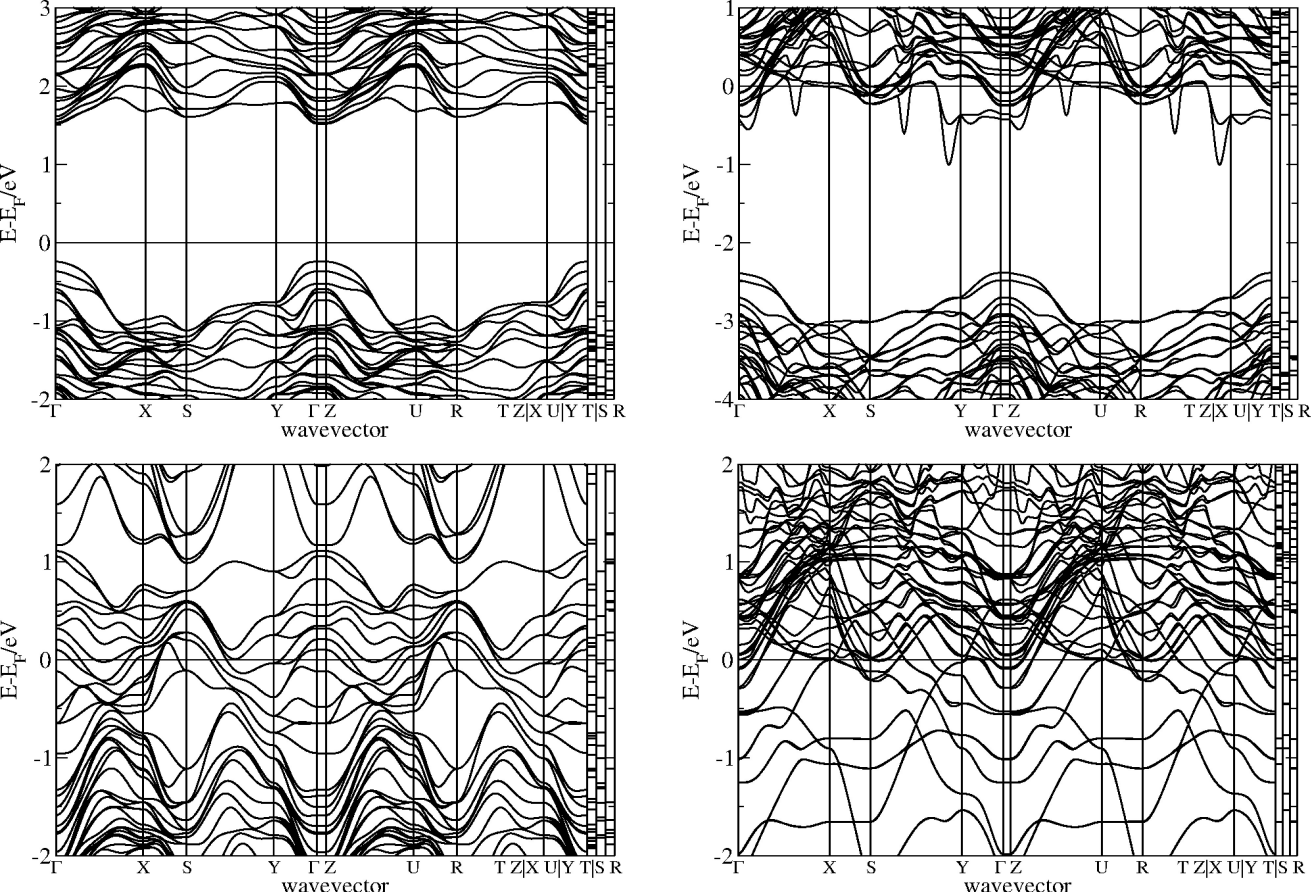
Band structures of ${\mathrm{TiO}}_{2}$ (left top), ${\mathrm{Nb}}_{1/3}{\mathrm{Ti}}_{2/3}O_{2}$ (right top), ${\mathrm{IrO}}_{2}$ (left bottom), ${\mathrm{RhO}}_{2}$ :${\mathrm{Nb}}_{1/3}{\mathrm{Ti}}_{2/3}O_{2}$ (right bottom).

In order to gain a deeper insight into the electronic properties, band structures were calculated and selected ones are displayed in Figure 8. The respective Fermi energy $E_{F}$ is subtracted from the band energies and the wavevector runs along the standard k‐point path for the symmetry of the ${\mathrm{TiO}}_{2}$ (110) surface obtained with SeeK‐path.^[76]^ Pure ${\mathrm{TiO}}_{2}$ shows a typical semiconductor band structure in which the Fermi energy is not intersected by any bands. In comparison with experimental values of 3.3^[77]^ or 3.6 eV^[78]^ the band gap is too small because hybrid functionals were not employed. This limitation is known and not focus of this work. Here, the analysis of qualitative trends between different structures takes priority. The band structures of all other investigated structures show metallic behavior with bands crossing the Fermi energy. From ${\mathrm{Nb}}_{1/3}{\mathrm{Ti}}_{2/3}O_{2}$ via ${\mathrm{IrO}}_{2}$ to ${\mathrm{RhO}}_{2}$ :${\mathrm{Nb}}_{1/3}{\mathrm{Ti}}_{2/3}O_{2}$ the number of bands crossing the Fermi level increases. At the same time more flat bands appear close to the Fermi energy. Conductivity and OER activity have been observed to correlate.^[27]^ This could indicate an increase of conductivity and fits with the results of the higher OER activity from the calculated overpotential.

## Conclusions

In the present work, we analyzed the oxygen evolution reaction on several rutile‐based materials, focusing on various reaction pathways corresponding to different intermediates. The adsorption is assumed to take place on the (110) surface with the thermodynamically most stable metal configuration. Pure rutile exhibits a poor activity due to the corresponding high adsorption energies, especially of M−O. By doping with 50 % niobium, this adsorption energy is strongly reduced. However, the improvement does not lead to the desired low overpotentials, in the present case approximated by adsorption energies of different intermediates. To improve the activity, doping and adding adlayers on pure and doped rutile is a strategy suggested by this work. The iridium‐based structures, either as the pure state‐of‐the‐art catalyst ${\mathrm{IrO}}_{2}$ or as adlayers on ${\mathrm{TiO}}_{2}$ and niobium‐doped rutile, have very similar overpotentials. Even though no activity improvement can be reached, the iridium percentage can be drastically reduced by using adlayers almost without increasing the overpotential. ${\mathrm{RuO}}_{2}$ has a lower overpotential as an adlayer on ${\mathrm{TiO}}_{2}$ ‐rutile compared to pure ${\mathrm{RuO}}_{2}$ , so the noble metal content can also be decreased while increasing the OER activity. By controlling which transition metal is available as the coordinatively unsaturated site, the activity can be tuned, which is shown for ${\mathrm{Nb}}_{1/3}{\mathrm{Ir}}_{1/12}{\mathrm{Ti}}_{7/12}O_{2}$ . In summary, two promising alternatives to pure ${\mathrm{IrO}}_{2}$ were identified here with lower overpotentials and thus better electrocatalytic properties. The structures with the lowest overpotential are ${\mathrm{RhO}}_{2}$ and ${\mathrm{RhO}}_{2}$ :${\mathrm{Nb}}_{1/3}{\mathrm{Ti}}_{2/3}O_{2}$ . The band structure of the latter shows more bands crossing the Fermi level than ${\mathrm{IrO}}_{2}$ . The other alternative to pure ${\mathrm{IrO}}_{2}$ is to use ${\mathrm{IrO}}_{2}$ only as an adlayer on another supporting material, like 33 % or 50 % niobium‐doped rutile.

## Computational Methods

For all DFT calculations presented here, the plane‐wave Vienna *ab initio* simulation package (VASP),[^79^, ^80^, ^81^, ^82^] version 6.4.3, the Perdew‐Burke‐Ernzerhof (PBE) functional^[50]^ and the projector‐augmented wave (PAW) approach[^83^, ^84^] were employed. The respective PAW parameters were extracted from the VASP POTCAR 5.4 files library: Ti 08Apr2002 (4 valence electrons (VE)), O 08Apr2002 (6 VE), H 15Jun2001 (1 VE), Nb_sv 17Jan2003 (13 VE), Ta 17Jan2003 (5 VE), V 08Apr2002 (5 VE), Ru 06Sep2000 (8 VE), Rh 06Sep2000 (9 VE), Sc_sv 07Sep2000 (11 VE), and Y_sv 06Sep2000 (11 VE). In order to take long‐range London dispersion into account the D3(BJ) dispersion correction[^57^, ^58^] was applied. For ${\mathrm{TiO}}_{2}$ , the D4 dispersion correction[^51^, ^52^] was also tested. The energy cut‐off was set to 450 eV for ${\mathrm{IrO}}_{2}$ , ${\mathrm{Ir}}_{0.5}{\mathrm{Ti}}_{0.5}O_{2}$ , ${\mathrm{IrO}}_{2}$ :${\mathrm{Ir}}_{1/3}{\mathrm{Ti}}_{2/3}O_{2}$ , and ${\mathrm{TiO}}_{2}$ with PBE−D3(BJ).[^50^, ^57^, ^58^] For the remaining substituted rutile structures, and ${\mathrm{TiO}}_{2}$ calculated with D4[^51^, ^52^] the cutoff was set to 900 eV, and for ${\mathrm{IrO}}_{2}$ :${\mathrm{TiO}}_{2}$ , ${\mathrm{IrO}}_{2}$ :${\mathrm{Nb}}_{0.5}{\mathrm{Ti}}_{0.5}O_{2}$ and ${\mathrm{Ir}}_{0.5}{\mathrm{Ti}}_{0.5}O_{2}$ both cutoffs were tried. The adsorption energies with the lower cutoff converged within 0.05 eV for ${\mathrm{IrO}}_{2}$ :${\mathrm{TiO}}_{2}$ , 0.07 eV for ${\mathrm{IrO}}_{2}$ :${\mathrm{Nb}}_{0.5}{\mathrm{Ti}}_{0.5}O_{2}$ and 0.04 eV for ${\mathrm{Ir}}_{0.5}{\mathrm{Ti}}_{0.5}O_{2}$ . For $H_{2}$ and $H_{2}O$ both cutoffs were compared. Total energies calculated with 450 eV differ 0.03 eV or less from ones calculated with 900 eV. Energy differences were calculated only from energies gained with the same cutoff. For geometry optimizations and the resulting adsorption energies the lower cutoff proved sufficient, yet for structures used for frequency calculations, 900 eV are neccessary. During the bulk geometry optimizations both atomic positions and lattice parameters were relaxed. Some bulk geometry optimizations yielded γ not being exactly 90°. Surfaces were constructed from these bulk structures but also from bulk structures with equal lattice parameters but a γ of 90°. The energy difference between these slightly asymmetric and symmetric slab models with γ =90° were negligible, which was shown by test calculations for Nb_0.5_Ti_0.5_O_2_. Thus, the other surfaces were constructed symmetrically to avoid artificial dipole moments. The vacuum distance of 12 Å has been used and proven sufficient in previous test calculations. For 50 % substitution of Nb, Ta, V, Sc, and Y, different metal atom configurations were tested. Compare Figures S1–S3 in Supporting Information for Sc, Y, and Nb as well as the 33 % substitution and for Nb_1/3_Ir_1/12_Ti_7/12_O_2_. During the optimization of the surface and adsorption models, the lattice parameters were kept constant and the atomic positions were relaxed. Six layers are sufficient to provide converged results for the (110) surface models. The surfaces consist of 6 layers based on the optimized bulk lattice parameters. For the addition of monolayers the bulk lattice parameters of the support materials were used. The number of layers are increased to eight and the metal atoms in the topmost layers were substituted by the respective dopant. Prior calculations of adsorbed oxygen species on pure TiO_2_‐rutile showed that only calculations with single oxygen atoms on the surfaces require an unrestricted Kohn‐Sham treatment. For all of the other structures except for antiferromagnetic RuO_2_ calculations without spin polarisation are sufficient. In the spin polarized calculations, the total magnetic moment was not fixed during SCF runs. The convergence criteria for the electronic self‐consistent cycle and forces were set to 10^−6^ eV and 0.01 eV/Å, respectively, for structure optimization. For the primitive unit cell (PUC) and the 2×2 supercell, the Monkhorst‐Pack grids were set to 8×4×1, for the 2×1 supercell to 4×4×1, and for the 3×1 to 4×6×1. Molecular hydrogen and H_2_O were calculated with 900 eV for the frequency calculations in a non‐cubic simulation box with a side length of 7 Å or larger. For vibrational frequencies, the convergence criteria for the electronic self‐consistent cycle was set to more accurate values, 10^−8^ eV . For all surface calculations except for pure TiO_2_, two inner layers were fixed during the vibrational calculations. Bader charges were calculated with the Bader Charge Analysis from the Henkelman Group.[^85^, ^86^, ^87^, ^88^, ^89^, ^90^] For the band structure calculation, the VASP2WANNIER90 interface^[75]^ was employed and the band structures were plotted with xmGrace, version 5.1.25.^[91]^ SeeK‐path from Materials Cloud^[76]^ was used to find the k‐point path for the surfaces in this work. *First‐principles* molecular dynamics simulations were performed employing the machine‐learning potential implemented in VASP.^[73]^ In order to reduce computational effort, a lower cutoff energy, 400 eV, was employed. The simulations were performed at 300 and 600 K for 100 ps with a timestep of 1 fs.

## Conflict of Interests

The authors declare no conflict of interest.

1

## Supporting information

As a service to our authors and readers, this journal provides supporting information supplied by the authors. Such materials are peer reviewed and may be re‐organized for online delivery, but are not copy‐edited or typeset. Technical support issues arising from supporting information (other than missing files) should be addressed to the authors.

Supporting Information

## Data Availability

The data that support the findings of this study are available in the supplementary material of this article.
