# Supplementary material for: Improving the OER Activity of Titania Via Doping and Adlayers
Source: ChemistryOpen. 2025 Feb 25;14(4):e202400085. doi: 10.1002/open.202400085 (PMC11973512; doi:10.1002/open.202400085)
Supplement: Supplementary file 1 — Supporting Information [file OPEN-14-e202400085-s001.pdf]

ChemistryOpen

Supporting Information

Improving the OER Activity of Titania Via Doping and Adlayers

Anna Gomer* and Thomas Bredow*

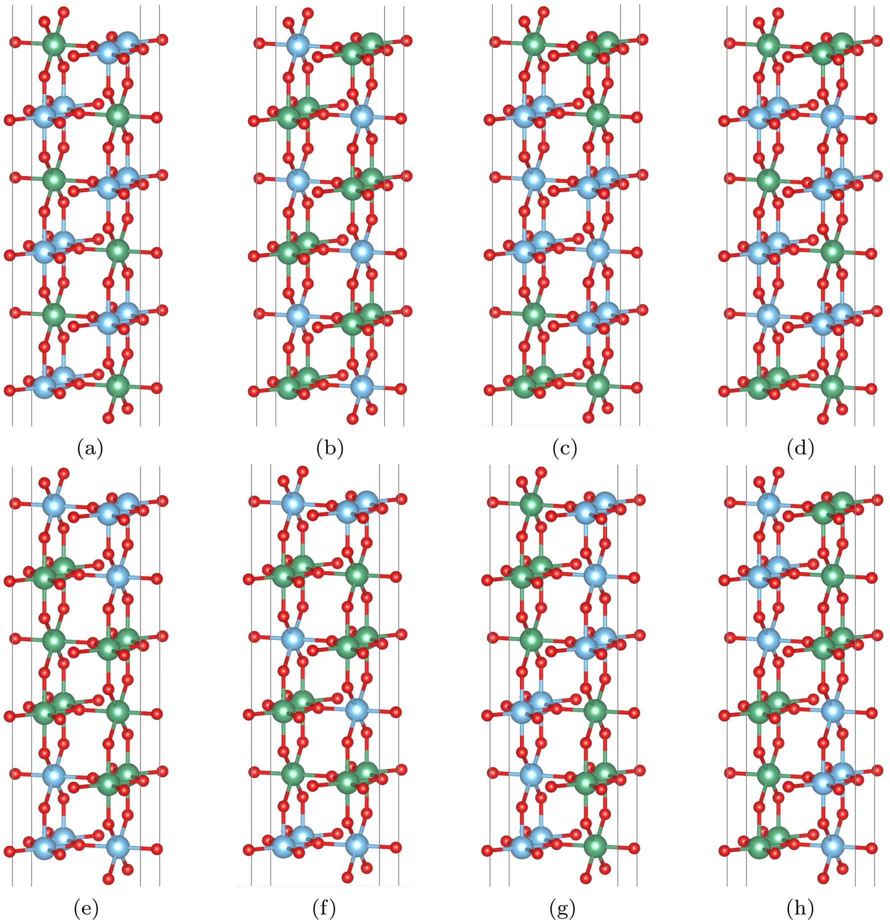

Figure S1: Metal cation configurations in the primitive unit cells of the doped TiO_2-rutile $M_{0.5}Ti_{0.5}O_2$ (110) surface with Ti as blue, M as green, and O as red spheres; visualized with VESTA [1].

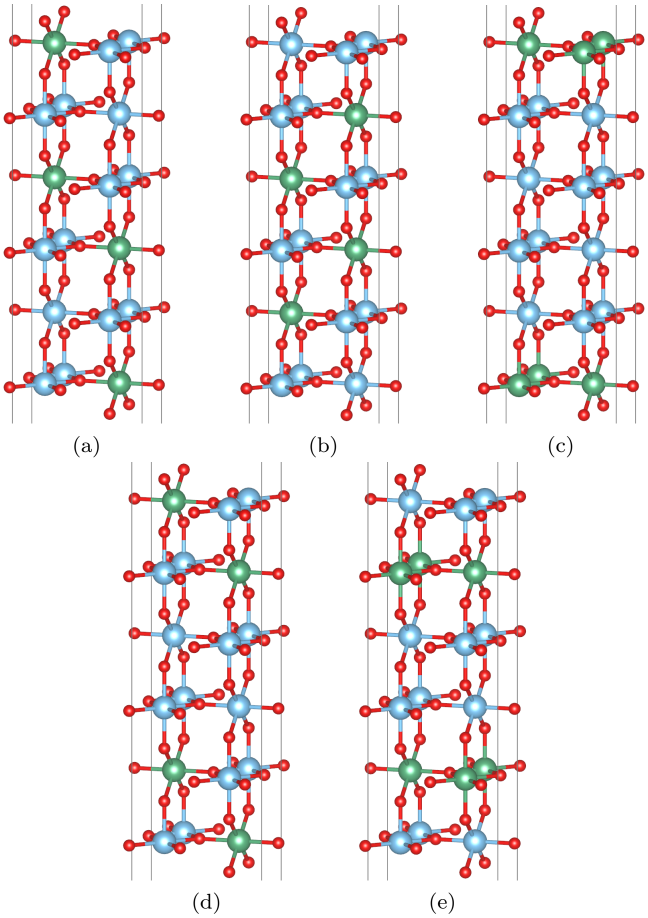

Figure S2: Metal cation configurations in the primitive unit cells of the doped TiO_2-rutile $M_{1/3}Ti_{2/3}O_2(110)$ surface with Ti as blue, M as green, and O as red spheres; visualized with VESTA [1].

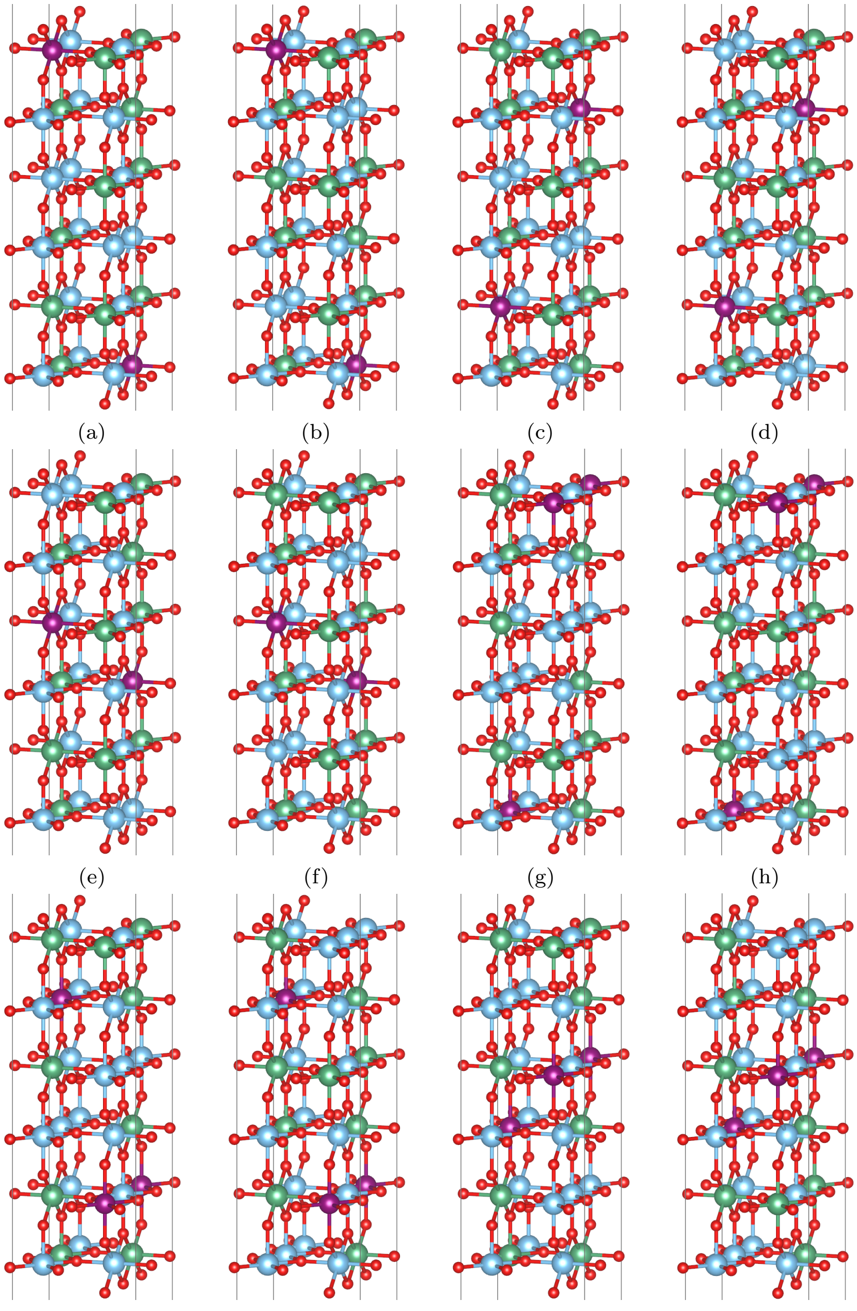

Figure S3: Metal cation configurations in the primitive unit cells of $Nb_{1/3}Ir_{1/12}Ti_{7/12}O_2(110)$ surface with Ti as blue, Nb as green, Ir as violet, and O as red spheres; visualized with VESTA [1].

References

[1] K. Momma and F. Izumi, "*VESTA3* for three-dimensional visualization of crystal, volumetric and morphology data," *Journal of Applied Crystallography*, vol. 44, no. 6, pp. 1272–1276, 2011.
